# Diversity and prevalence of dairy streptococcal phages in two European dairy fermentation facilities over a one-year period

**DOI:** 10.1099/mgen.0.001729

**Published:** 2026-05-26

**Authors:** Kelsey White, Joanna Danuta Szafran, Brian McDonnell, Gargee Chawande, Louisa Faherty-McGrath, Gabriele Andrea Lugli, Marco Ventura, Giovanni Eraclio, Federica Volonté, Fabio Dal Bello, Douwe van Sinderen, Jennifer Mahony

**Affiliations:** 1 School of Microbiology & APC Microbiome Ireland, University College Cork, Western Road,, Cork T12 YT20, Ireland; 2 Laboratory of Probiogenomics, Department of Chemistry, Life Sciences, and Environmental Sustainability, University of Parma, 12 - I 43121 Parma, Italy; 3 Sacco Srl, Via Manzoni 29/A, 22071 Cadorago (Co), Italy

**Keywords:** bacteriophage, dairy, longitudinal, phageome, receptor binding, *Streptococcus thermophilus*

## Abstract

Bacteriophages (or phages) represent a major challenge to industrial dairy fermentations and may cause reduced acidification of the milk substrate. *Streptococcus thermophilus* is a thermophilic lactic acid bacterium widely used in dairy fermentations. Here, we sought to establish the presence and diversity of phages present in two distinct factories employing the same dairy streptococcal starter cultures, employing a culture-based phage screening approach supplemented with culture-independent phageome analyses. It was established that cheese whey samples from one factory had a very low abundance/diversity of phages, while those of the second factory contained much higher levels and diversity of phages. In total, 41 phages were isolated, and among these were 34 *Moineauvirus* and 7 *Vansinderenvirus* members. Phylogenetic analysis of the deduced receptor-binding proteins of these phages established homogeneity among those of *Vansinderenvirus* and significant heterogeneity among *Moineauvirus* members. Furthermore, the receptor-binding protein phylogeny data combined with host range and genotyping data indicate that the receptor for both *Vansinderenvirus* and *Moineauvirus* phages is a carbohydrate, which creates a roadmap for further investigation into the binding preferences of these industrially significant phages.

## Data Summary

Raw virome sequences are available at the National Center for Biotechnology Information Sequence Read Archive (https://www.ncbi.nlm.nih.gov/sra) under the BioProject accession number PRJNA1377139 (individual BioSample accession numbers are included in Table 1). The genome sequences of the phages sequenced within this study have been deposited in the GenBank database (https://www.ncbi.nlm.nih.gov/genbank/) under the accession numbers PX138259–PX138262 and PX138264–PX138300 (individual accession numbers for each genome can be found in Table 2).

**Impact Statement**

*Streptococcus thermophilus* strains are extensively used in thermophilic dairy fermentations; infection of these starter strains by bacteriophages remains one of the most significant threats to successful dairy fermentations. Close monitoring of phage populations present in fermentation settings is essential to circumventing the ever-persisting phage threat. In the current study, the phage populations present in whey samples collected from an Italian and Austrian cheese factory were assessed over a 13-month period using both culture-dependent phage screening and isolation, as well as virome sequencing and downstream phageome analysis. This ultimately led to the isolation and genomic sequencing of 41 dairy streptococcal phages encompassing 2 genera (*Moineauvirus* and *Vansinderenvirus*). Subsequent genomic analysis allowed for further insights into the diversity of proteins comprising the phage adhesion device and possible phage-encoded counter-defence systems. Additionally, phageome analysis led to the identification of persistent phage populations in the Italian factory, highlighting the usefulness of combining culture-dependent and -independent approaches to defining phage populations in dairy fermentation settings.

## Introduction

Bacteriophages (or phages) are viruses that specifically infect strains of a given bacterial species. In dairy fermentations, phage infection of lactic acid bacterial starter cultures is a consistent and potentially devastating threat, underpinning decades of research focused on dairy phage–host interactions [1,6]. Dairy fermentation facilities have adopted several strategies to reduce the risk of phage infection of starter cultures, including thermal treatment of milk, sanitization regimes and starter culture selection and/or rotation [7,9]. Despite these efforts, phages remain a persistent challenge in dairy fermentation facilities. Indeed, phage ecology studies have highlighted the persistence of phages in dairy fermentation factories over extended periods of time [10,13]. In cases where highly complex, so-called undefined starter culture systems are applied, phage diversity in cheese wheys is higher when compared to defined starter cultures, such as those used in yoghurt production, which exhibit much lower complexity [10]. While phages may be introduced into commercial dairy fermentation facilities through raw milk [14,15] or other ingredients, the repeated application of preferred/specific starter cultures appears to dictate the abundance and prevalence of a given phage in a factory [12,16].

*Lactococcus lactis*, *Lactococcus cremoris* and *Streptococcus thermophilus* are the most widely exploited and industrially significant bacterial species applied in dairy fermentations globally. Phages infecting these species have been classified into ten (for the two *Lactococcus* species) and five genetically distinct genera, respectively [17,18]. Among dairy streptococcal phages, members of the *Moineauvirus* and *Brussowvirus* genera (previously termed the *cos* and *pac* group phages, respectively) are reported as the most frequently encountered in thermophilic dairy fermentations [19,25]. However, more recent studies have highlighted the emergence of novel phage genera that are increasingly prevalent in global fermentation facilities, i.e. those belonging to the *Vansinderenvirus* (formerly termed the 5093 phages), *Piorkowskivirus* (formerly termed the 987 phages) and the P738 phage group [26,29]. These findings have reinforced the need for consistent phage monitoring to determine the diversity, prevalence and abundance of resident phage in order to develop and implement appropriate phage control measures.

Several dairy streptococcal phages have been described to infect a particular host strain through the recognition and binding to a saccharidic receptor on the host cell surface, mediated by the phage-encoded bona fide receptor-binding protein [25,30,32]. This saccharidic receptor represents either (part of) the cell wall-associated rhamnose-glucose polysaccharide (RGP) or the exopolysaccharide (EPS). It has been proposed, but not yet experimentally verified, that EPS is the receptor for both *Moineauvirus* and *Vansinderenvirus* phages [26,33]. Additionally, it has been proposed that certain *Brussowvirus* members recognize RGP [34], while it has been experimentally demonstrated that *Piorkowskivirus* and P738 phages recognize EPS and RGP, respectively [32,35]. The carbohydrate composition and structure of these complex glycans are highly variable among dairy streptococci, which underpins the narrow host range of most dairy streptococcal phages [30,36, 37]. Phage susceptibility of a given *S. thermophilus* strain is further influenced by the specific repertoire of antiphage systems that it harbours. The phage defensome of *S. thermophilus* is dominated by clustered regularly interspaced short palindromic repeats (CRISPR) and restriction/modification (R/M) systems and enhanced by additional strain-specific systems [38,41]. It has previously been demonstrated that CRISPR-Cas and R/M systems work cooperatively to enhance phage resistance of *S. thermophilus* [42,43].

In response to the pressure exerted by anti-phage systems, dairy streptococcal phage genomes have acquired genes encoding methyltransferases and anti-CRISPR systems [44,46]. Three anti-CRISPR (Acr) systems have been identified to date within the genomes of *S. thermophilus*-infecting phages, i.e. AcrIIA3, AcrIIA5 and AcrIIA6, which inhibit one or more of the dominant CRISPR-Cas systems of this species [45,47]. Given the genetic diversity of dairy streptococcal phages and the number of uncharacterized genes within their genomes, it is likely that additional counter-defence systems are yet to be discovered. Therefore, it is essential to continuously assess and define phage populations present in dairy fermentation facilities to understand the abundance, prevalence, diversity and persistence of phages to mitigate the risk of fermentation failure or product inconsistencies. In the current study, we evaluated phages present in whey samples from an Italian and Austrian cheese factory over a 13-month period by culture-based phage isolation methods and virome (all virus-like particles and corresponding DNA within a sample) sequencing with downstream phageome (assembled phage-like genetic components) analysis. These efforts led to the isolation of 41 distinct phages that were used to evaluate genetic diversity and host range, with particular focus on proteins comprising the phage adhesion device and possible phage counter-defence systems.

## Methods

### Bacterial strains and phages

In total, 58 industrial strains of *S. thermophilus* were employed in this study (Table S1, available in the online Supplementary Material). Of these strains, 23 strains form a ‘core’ set of strains (indicated in Table S1) that were used in starter cultures in both the Italian and Austrian factories from which whey samples were sourced, while the additional 35 strains had been employed in one of the factory sampling sites (but not the other) at least once across the sampling period. Strains were routinely grown overnight at 42 °C from single colonies or bacterial stocks maintained at −70 °C [25% *w/v* glycerol in M17 broth (Millipore Sigma-Aldrich, Gillingham, UK) supplemented with 1% lactose (LM17) and 0.5% yeast extract (Becton, Dickinson and Company, USA)]. Phages (derived from either single plaques or phage lysates) were propagated on appropriate host strains in LM17 broth supplemented with 10 mM CaCl_2_ (Millipore Sigma-Aldrich) and incubated at 42 °C until lysis was observed.

### Host strain genotyping

*S. thermophilus* strains used in this study were typed using colony PCR employing previously described dual *rgp* genotyping multiplex primers based on the variable (V) and backbone (B) regions of the *rgp* gene cluster [30].

### Bacteriophage screening and isolation from whey samples

Eighty-five whey samples (Table S2) sourced from two dairy fermentation facilities were analysed in this study for the presence of phages capable of infecting one or more strains from the core panel of 23 *S*. *thermophilus* strains (i.e. strains present in starter cultures utilized in both factories, Table S1) used in thermophilic dairy fermentation factories where delayed milk acidification was observed, indicating phage issues. Whey samples were centrifuged at 4,500 ***g*** for 20 min at 4 °C to remove particulates; the resulting supernatant was subsequently filtered through a 0.45-µm filter (Sarstedt, Nümbrecht, Germany) and stored at 4 °C until use. Phage screening was performed using the double agar spot assay method [48], using LM17 supplemented with 10 mM CaCl_2_, 0.5% glycine (Millipore, Sigma-Aldrich) and 1% (solid) or 0.4% (semi-solid) agar (Millipore, Sigma-Aldrich). Ten microlitres of the processed/filtered whey samples was spotted on individual soft agar overlays containing 400 µl of each of the 23 core *S. thermophilus* strains. Phages from lysis-positive samples were subsequently purified by plaque assays. Phages were propagated (initially from a single plaque) on appropriate host strains (by adding phage concurrently with inoculation) in LM17 broth supplemented with 10 mM CaCl_2_ and incubated at 42 °C until lysis had occurred. Phage lysates were filtered (0.45 µm) and stored at 4 °C.

### Host range analysis

The host range of isolated phages was determined using a panel of 58 *S*. *thermophilus* strains (see above and Table S1) in order to identify secondary hosts for the identified phages. This was performed using spot assays, where phage lysate (following propagation on primary host) dilutions were spotted onto LM17 double-layer agar plates as described above and confirmed by plaque assays. All assays were performed in triplicate.

### Virome DNA extraction, sequencing and annotation

Viral particles were isolated from a selection of seven whey samples representing early, mid and late sampling points from the Italian and Austrian factories at months 1–2, 6 and 12 (samples S1, S13, S34, S36, S85, S86 and S87, Table S2), using a previously described viral extraction method with minor modifications, as follows: 10 ml of whey was centrifuged initially at 300 ***g*** for 5 min (4 °C). NaCl (Millipore, Sigma-Aldrich) was then added to a final concentration of 1 M to the supernatant and subsequently incubated at 4 °C for at least an hour. The pH of the whey samples was then adjusted to ~4.6 using 0.1 M HCl (Millipore, Sigma-Aldrich) or 0.1 M NaOH (Millipore, Sigma-Aldrich) and then centrifuged at 28,000 ***g*** for 15 min (4 °C). The resulting supernatant was filtered first with a 0.45-µm filter, followed by a 0.2-µm filter (Sarstedt). Viral particles of this whey filtrate were then precipitated with PEG 6000 (Millipore, Sigma-Aldrich) at a final concentration of 10% (w/v) overnight at 4 °C. Samples were then centrifuged at 15,000 ***g*** for 15 min (4 °C), after which the resulting pellet was resuspended in 1 ml SM buffer (50 mM Tris-HCl, 100 mM NaCl, 10 mM MgSO_4_ and 10 mM CaCl_2_ [49]). The suspension was then treated with an equal volume of chloroform, mixed vigorously and then centrifuged at 5,000 ***g*** for 5 min. The aqueous layer was transferred to a separate tube, and a viral DNA extraction procedure was performed using the Norgen Biotek Phage DNA isolation kit (Norgen Biotek, Thorold, ON, Canada), according to the manufacturer’s instructions.

DNA sequencing libraries were prepared using the Nextera XT DNA Library preparation kit (Illumina, CA, USA), following the manufacturer’s guidelines. One nanogram of DNA was used for library preparation undergoing fragmentation and amplification. Paired-end (300 cycles, 2×150 bp) sequencing was performed by GenProbio srl (Parma, Italy) on a NextSeq platform (Illumina). Phageomes were assembled using two different assembly and associated annotation pipelines/methods: METAnnotatorX2 and Phables. Filtered FASTQ files were used to assemble and annotate phage contigs using the METAnnotatorX2 pipeline [50]. The bioinformatic tool Phables was additionally used to resolve phage genomes from viral metagenomic assemblies (generated using the open-source assembler SPAdes v3.15.4 [51]) as previously described [16,52]. Phables-assembled phage contigs were annotated using Pharokka v1.7.5 [53]. Taxonomic classification of the assembled phage contigs was performed by the respective annotation pipelines as previously described [50,53].

### Phage DNA extraction, sequencing and annotation

Phage DNA was extracted from 1 ml of fresh phage lysate (>10^7^ p.f.u. ml^−1^) using the Norgen Biotek Phage DNA isolation kit as per the manufacturer’s protocol. The resulting genomic DNA was stored at −20 °C until required. Phage genome sequences were determined by GenProbio srl (Parma, Italy) using a MiSeq platform (Illumina, San Diego, CA, USA) according to the supplier’s protocol. Phage genome libraries were prepared using an Illumina Nextera XT DNA Library Preparation Kit (Illumina Inc.). Libraries were quantified using a fluorometric Qubit quantification system (Life Technologies, USA) and loaded on a 2200 TapeStation instrument (Agilent Technologies, USA). Sequencing was performed using the Illumina MiSeq platform with a 600-cycle flow cell version 3 (Illumina Inc.). Fastq files of Illumina paired-end reads (250 bp) obtained from phage sequencing efforts were used as input for genome assembly through the MEGAnnotator2 pipeline [54]. The SPAdes program v3.14 was used for *de novo* assembly of phage sequences with the pipeline option ‘--careful’ and a list of k-mer sizes of 21, 33, 55, 77, 99 and 127 [51]. Contigs were then employed by MEGAnnotator2 for the prediction of protein-encoding ORFs using Prodigal [55]. Predicted ORFs were functionally annotated by means of DIAMOND against the National Center for Biotechnology Information (NCBI) RefSeq database and INTERPRO [56] against the PFAM database. Furthermore, tRNA genes were identiﬁed using tRNAScan-SE version 2.0 [57].

### Phageome reads mapping analysis using CoverM

A phage database was established using publicly available complete dairy streptococcal and lactococcal phage sequences retrieved from the NCBI viruses (https://www.ncbi.nlm.nih.gov/labs/virus/), sorted according to their genera (ten lactococcal and five dairy streptococcal genera) as previously described [16]. The relative abundance of members belonging to each of these phage genera present in each virome sample was then determined using CoverM v0.4.0 (https://github.com/wwood/CoverM; ‘genome’ mode) with a minimum read percent identity threshold of 80% and a minimum read aligned percentage of 50%. Additionally, the relative abundance of streptococcal receptor-binding protein (RBP)-encoding genes present in a given virome sample was determined by mapping a representative RBP-encoding nucleotide sequence of each *S. thermophilus* phage genus or group/subgroup (in the case of moineauviruses and brussowviruses, which will be defined below) using CoverM (‘contig’ mode). This was achieved by calculating the reads per kb of exon per million using the following parameters, in order to account for: read percent identity >70%, read coverage >90% and covered fraction >90%. Cutoffs were determined based on minimum identity thresholds of the established RBP groups/subgroups (which will be defined below). Reads mapping was performed in order to determine the presence or absence of closely related streptococcal phages present in the whey viromes. To achieve this, CoverM (‘contig’ mode) was used to calculate the covered fraction of the sequenced *S. thermophilus* phage genomes, with a percent identity cut-off of 85% and coverage minimum of 95%, and only phage genomes for which >90% of their length was covered are reported.

### Comparative genomic analysis of streptococcal phage genomes and phageome-derived contigs

The genome sequences of the isolated streptococcal phages were compared to publicly available genomes through blastn analysis (using default parameters) to determine their genus assignment (based on the top hit). Multiple alignment of the nucleotide sequences (or protein sequences) of phage genomes or their encoded proteins was performed using clustalw software (using default parameters). The alignment was employed to generate both rooted and unrooted phylogenetic trees using the iTOL v6 software (https://itol.embl.de/). Phage genome alignments were generated using the CAGECAT version 1.0 web platform (https://cagecat.bioinformatics.nl/tools/clinker) [58]. Comparisons of the sequenced phage genomes and individual phage genes/encoded proteins of interest were performed by all-against-all, bi-directional blast alignment using default parameters [59]. REBASE (http://rebase.neb.com/rebase/rebase.html) was used to analyse putative phage-encoded methyltransferases (MTases) [60]. Hadamard scores, which represent a quantitative evaluation of the average nucleotide identity (ANI) and coverage between DNA sequences, were calculated using the ANIm method from pyANI (https://github.com/widdowquinn/pyani) [61] in order to assess the genetic diversity between sequenced streptococcal phage genomes.

Streptococcal phage contigs derived from phageome assemblies were selected for further genomic comparisons (using blast as described above) to sequenced phage genomes on the basis of a >25.0 kbp size cutoff, ensuring a particular contig constituted a complete or at least nearly complete phage genome [62].

### Bioinformatic analysis of phage-encoded adhesion proteins

The head domains of representatives of streptococcal phage genera were determined using HHpred (Homology detection and structure prediction by HMM-HMM comparison) [63] employing default settings. Structural predictions of the carbohydrate-binding domains within the tail-associated lysin (Tal) of representative moineauviruses were performed by AlphaFold3 via Google servers (https://golgi.sandbox.google.com) [64]. Visual representation of the final predicted domain structures was prepared with ChimeraX [65] and then submitted to the Dali server to identify closest structural homologs in the protein database [66].

## Results

### Isolation of streptococcal phages from various dairy fermentations

In the current study, whey samples derived from two factories that utilize the same thermophilic starter cultures (in addition to starter cultures provided by other suppliers) in Italy and Austria to produce mainly mozzarella and pizza cheese (typically from pasteurized milk) were evaluated for the presence and diversity of phages over a period of 13 months (13 sampling points across October 2022–October 2023). In total, 85 whey samples were collected (Italy *n*=47; Austria *n*=38) from these two factories. Samples were collected monthly to facilitate a longitudinal and comparative analysis. Whey samples were screened against a panel of 23 core strains that are common to the starter culture blends used in both of the above-mentioned factories. To establish the genetic diversity among members of the core panel of 23 *S*. *thermophilus* strains, their *rgp* genotypes were identified by a two-step multiplex PCR. Through this approach, it was established that the panel of strains included five V1B1, nine V1B2, two V3B2 and seven V3B3 *rgp* genotype strains (Table S1).

Phage titres in the whey samples on the sensitive host strains ranged between 10^2^ and 10^8^ p.f.u. ml^−1^ with a typical titre of 10^5^ p.f.u. ml^−1^. In total, 1,955 spot assays were performed (85 whey samples × 23 *S*. *thermophilus* strains), and in this manner, it was determined that 42 of the samples (49%) harbour phages capable of infecting at least one strain in the core test panel. Seven of the 23 core strains were not sensitive to phage infection, while 16 strains were identified as susceptible to infection by at least one phage. To derive an understanding of the phage sensitivity of the broader panel of strains as a ‘risk evaluation’ approach, the 85 whey samples were subsequently tested against an additional 35 strains that were used on occasion (but not routinely) in 1 or the other factories. This allowed us to establish the number of strains that were infected across the sampling period and to develop a basis for the selection of phage–host combinations to attempt propagations (Table 1).

**Table 1.** Summary of phage screening efforts of the 85 cheese whey samples collected from dairy fermentation factories in Italy and Austria against a panel of 58 S. *thermophilus* strains

Sample month	No. strains infected	Phage-containing sample	No. phages selected for genome sequencing	Phage name
Oct. 2022 (month 1)	6	S1, S2, S4	2	P01-P02
Nov. 2022 (month 2)	3	S9, S10, S12	4	P03-P06
Dec. 2022 (month 3)	7	S16, S17	1	P07
Jan. 2023 (month 4)	2	S23, S24	0	–
Feb. 2023 (month 5)	4	S28, S29, S31	0	–
Mar. 2023 (month 6)	4	S35–S37	0	–
Apr. 2023 (month 7)	10	S41–S44	5	P08-P12
May 2023 (month 8)	4	S48, S49	1	P13
June 2023 (month 9)	6	S55–S57	0	–
July 2023 (month 10)	13	S62–S65, S68	0	–
Aug. 2023 (month 11)	15	S69–S72, S75	8	P15-P22
Sept. 2023 (month 12)	21	S79–S82	11	P23-P33
Oct. 2023 (month 13)	6	S86, S87, S89	9	P34-P42

Based on the spot assay data, 97 distinct phage-host interactions were identified (Table S3), and interestingly, phage screening of the whey samples against the panel of *S. thermophilus* strains identified only one phage isolate from the Austrian factory. However, only 41 of the 97 distinct phage–host combinations achieved a sufficient titre (>10^5^ p.f.u. ml^−1^ where the host strains produce discernible/concerted lawns in plaque assays) to facilitate further analysis. Among the 41 phages, 25 were isolated on a strain presenting a variable 1 (V1) genotype and 16 infected those with a V3 genotype.

### Repeated starter culture application facilitates circumvention of phage-resistant strains

**Fig. 1.**
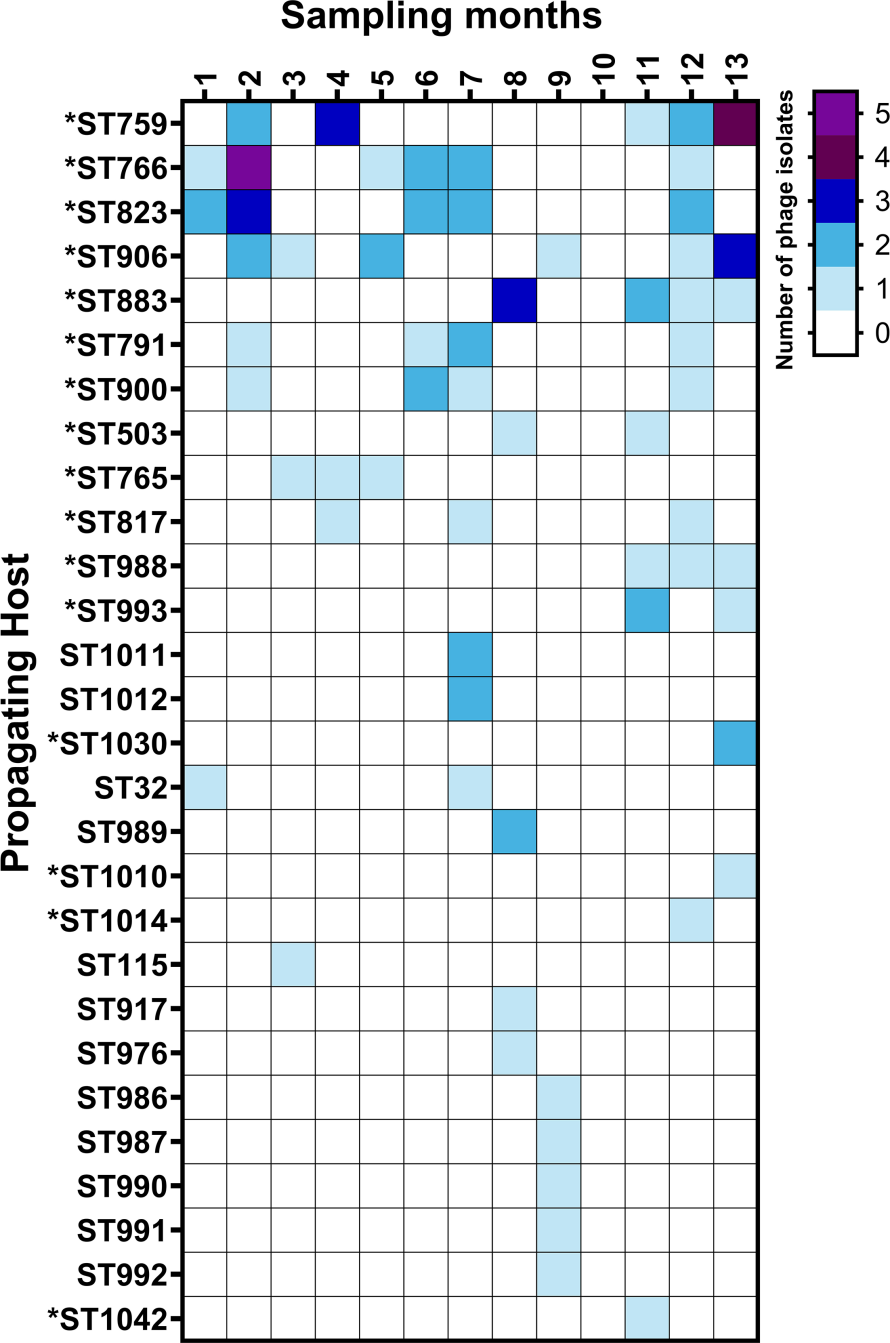
Phage screening results of whey samples originating from an Italian and Austrian fermentation factory employing the same dairy streptococcal starter cultures against a panel of *S. thermophilus* strains. *S. thermophilus* strains belonging to the core strain panel are indicated by an asterisk. The number of phage isolates identified against a particular strain at a specific sampling month is indicated by coloured squares as depicted in the figure legend. The absence of an identified phage isolate is indicated by white squares.

The Austrian factory-derived whey samples appeared to contain a very limited number of phages against the panel of strains in our collection. In contrast, whey samples originating from the Italian factory were shown to contain an abundance of phages across the sampling period against 16 of the 23 core strains. Among these susceptible strains, seven appeared to be particularly vulnerable to phage infection (i.e. ST759, ST766, ST823, ST906, ST883, ST791 and ST900). These strains were infected by phages present in multiple whey samples across the 13-month period (Fig. 1, Table S3) with a notable increase in the number of phage-sensitive strains across the study period. For example, in the first 6 months of the study, the number of strains infected by phages in the whey samples ranged between 2 and 7, while in the latter half of the year, the number of strains infected rose to between 5 and 21, with a most notable increase in months 10, 11 and 12 (Table 1). This increase in the number of strains that were shown to be phage sensitive in the latter sampling months can in part be explained by circumvention of bacteriophage-insensitive mutants (BIM) of parental strains that are also part of the core strain panel, e.g. ST1010 (BIM of ST759), ST1014 (BIM of ST823), ST1030 (BIM of ST906) and ST1042 (BIM of ST115). For example, the BIM ST1042 was shown to be sensitive to infection by phages present in a whey sample from month 11, while phages emerged against BIMs ST1014 in the month 12 samples and against ST1010 and ST1030 in the final sampling month. However, the circumvention of BIMs does not fully account for the increase in the number of sensitive host strains (as additional, non-BIM strains were also increasingly infected) and suggests an increased diversity of phages present in this factory across the sampling period. Therefore, phages that (i) infected (multiple) highly sensitive host strains (strains ST759, ST766, ST823, ST906, ST883, ST791 and ST900) across the sampling period, (ii) represented unique phage-host relationships and/or (iii) represented phages from early, mid- or late sampling time points were selected for genome sequence analysis. Across the 13-month period (October 2022 to October 2023 inclusive), 41 representative phages were propagated on their respective hosts and their genomes sequenced (Table 1).

### *Moineauvirus* and *Vansinderenvirus* members dominate the phage population

The genomes of 41 representative phages were sequenced to completion and compared to define the population complexity and intra-genus diversity. Among the 41 phages, 40 were isolated from the Italian factory analysed in this study. Based on comparative genome and blastn analyses, 6 and 34 of these 40 phages were identified as members of the *Vansinderenvirus* and *Moineauvirus* genera, respectively. The remaining phage whose genome was sequenced was isolated from the Austrian factory and was identified as a *Vansinderenvirus*. The genomes of the *Vansinderenvirus* isolates ranged between 32.1–34.0 kb in size, while those of the *Moineauvirus* isolates were 33.7–39.2 kb in length (Table 2), being consistent with the literature [33,44].

**Table 2.** Characteristics of streptococcal phages isolated and sequenced from whey samples employed in the present study

Phage	Phage group	Cluster # (*Moineauvirus*)	Host (*rgp*)	Factory origin	Whey sample	Genome size (bp)	No. ORF	GC%	GenBank accession #
P01	*Vansinderenvirus*		ST766 (V1B1)	Italy	S1	32130	40	38.29	PX138269
P02	*Vansinderenvirus*		ST32 (V1B2)	Italy	S2	33986	43	37.84	PX138280
P03	*Moineauvirus*	2	ST900 (V1B1)	Italy	S11	38524	47	38.23	PX138291
P04	*Moineauvirus*	2	ST766 (V1B1)	Italy	S12	36531	41	38.81	PX138295
P05	*Moineauvirus*	2	ST823 (V1B2)	Italy	S12	33705	41	39.09	PX138296
P06	*Moineauvirus*	2	ST906 (V3B3)	Italy	S12	35032	43	39.12	PX138297
P07	*Moineauvirus*	2	ST115 (V1B1)	Italy	S17	36521	41	38.69	PX138298
P08	*Moineauvirus*	3	ST823 (V1B2)	Italy	S41	34319	41	38.66	PX138299
P09	*Moineauvirus*	2	ST766 (V1B1)	Italy	S42	35638	39	39.02	PX138300
P10	*Moineauvirus*	3	ST817 (V1B2)	Italy	S42	34325	41	38.66	PX138259
P11	*Vansinderenvirus*		ST32 (V1B2)	Italy	S43	34030	43	37.9	PX138260
P12	*Moineauvirus*	2	ST817 (V1B2)	Italy	S43	34303	42	38.91	PX138261
P13	*Moineauvirus*	2	ST765 (V1B1)	Italy	S49	34710	38	38.84	PX138262
P15	*Moineauvirus*	1	ST759 (V3B2)	Italy	S70	35217	40	38.81	PX138264
P16	*Moineauvirus*	3	ST883 (V3B3)	Italy	S70	35024	42	38.19	PX138265
P17	*Moineauvirus*	2	ST906 (V3B3)	Italy	S70	36223	41	38.79	PX138266
P18	*Moineauvirus*	3	ST503 (V1B2)	Italy	S71	35022	43	38.21	PX138267
P19	*Moineauvirus*	2	ST503 (V1B2)	Italy	S71	34063	38	39.03	PX138268
P20	*Moineauvirus*	2	ST1042 (V1B1)	Italy	S71	36301	41	38.98	PX138270
P21	*Moineauvirus*	2	ST883 (V3B3)	Italy	S72	36382	41	38.79	PX138271
P22	*Moineauvirus*	2	ST988 (V1B2)	Italy	S72	35165	40	38.89	PX138272
P23	*Moineauvirus*	1	ST759 (V3B2)	Italy	S79	34693	40	38.9	PX138273
P24	*Moineauvirus*	2	ST817 (V1B2)	Italy	S79	34986	41	38.82	PX138274
P25	*Vansinderenvirus*		ST759 (V3B2)	Italy	S80	33096	40	38.18	PX138275
P26	*Moineauvirus*	2	ST817 (V1B2)	Italy	S80	34691	41	38.88	PX138276
P27	*Moineauvirus*	3	ST823 (V1B2)	Italy	S80	35516	42	38.51	PX138277
P28	*Moineauvirus*	3	ST883 (V3B3)	Italy	S80	33988	40	38.33	PX138278
P29	*Moineauvirus*	2	ST906 (V3B3)	Italy	S80	34702	41	39.01	PX138279
P30	*Moineauvirus*	2	ST766 (V1B1)	Italy	S81	36545	42	38.89	PX138281
P31	*Moineauvirus*	3	ST823 (V1B2)	Italy	S81	35388	42	38.51	PX138282
P32	*Moineauvirus*	3	ST1014 (V1B2)	Italy	S81	35380	42	38.52	PX138283
P33	*Moineauvirus*	2	ST900 (V1B1)	Italy	S82	39175	48	38.48	PX138284
P34	*Vansinderenvirus*		ST759 (V3B2)	Austria	S85	33093	41	38.13	PX138285
P35	*Moineauvirus*	1	ST759 (V3B2)	Italy	S86	36731	41	38.64	PX138286
P36	*Moineauvirus*	2	ST988 (V1B2)	Italy	S86	35739	40	39.01	PX138287
P37	*Moineauvirus*	2	ST1030 (V3B3)	Italy	S87	34272	44	38.96	PX138288
P38	*Moineauvirus*	1	ST759 (V3B2)	Italy	S88	34592	40	38.89	PX138289
P39	*Vansinderenvirus*		ST759 (V3B2)	Italy	S89	33022	41	38.14	PX138290
P40	*Moineauvirus*	1	ST883 (V3B3)	Italy	S89	34710	38	38.88	PX138292
P41	*Moineauvirus*	2	ST993 (V1B1)	Italy	S89	35997	41	38.96	PX138293
P42	*Vansinderenvirus*		ST1010 (V3B2)	Italy	S89	32971	40	38.15	PX138294

**Fig. 2.**
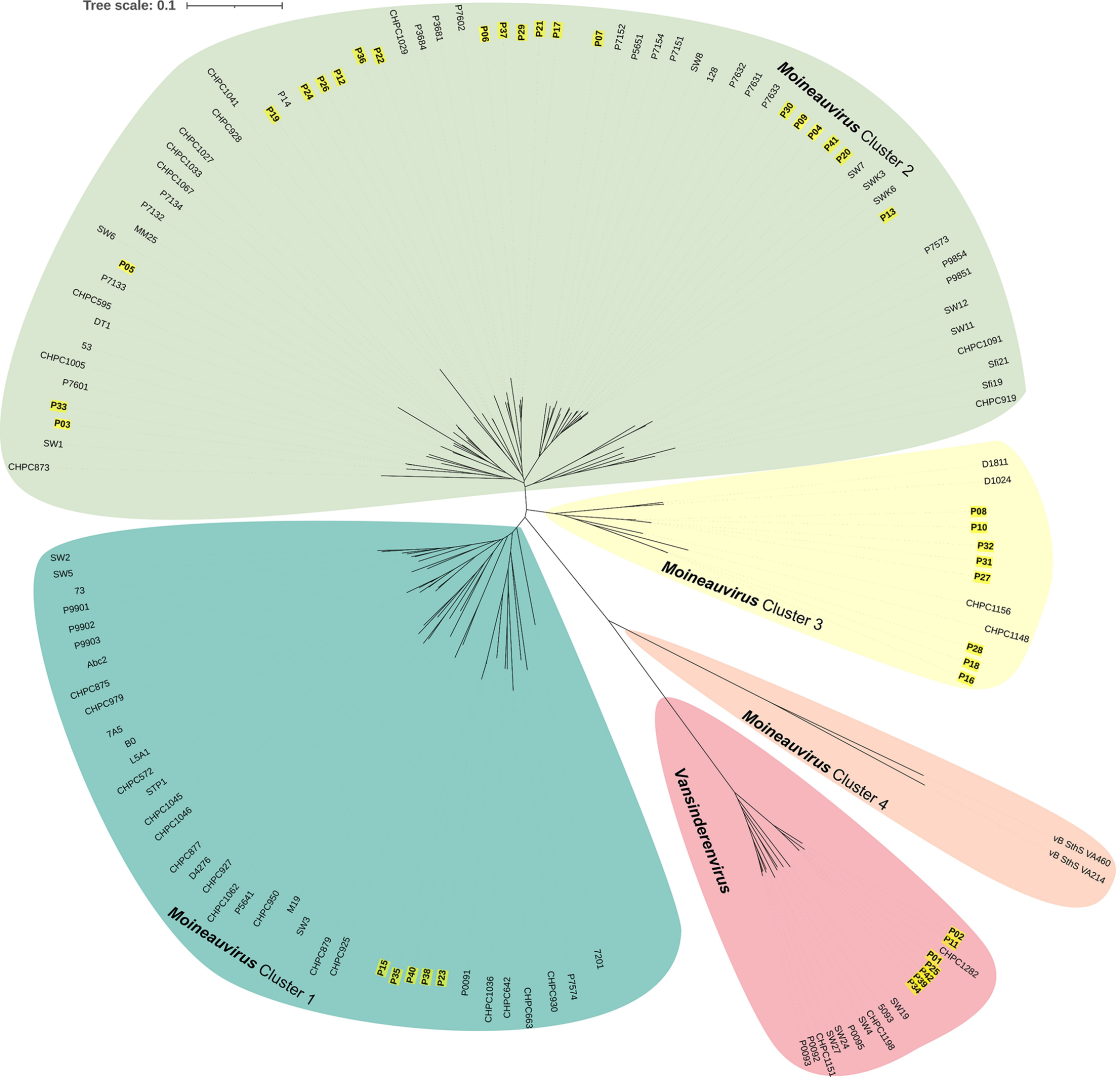
Unrooted phylogenetic tree of the 41 sequenced streptococcal phage genomes alongside complete genomes of publicly available *Moineauvirus* and *Vansinderenvirus* representatives (93 in total) retrieved from the NCBI database. Genomes of phages that were sequenced in the context of this study are indicated in yellow-highlighted text. The *Moineauvirus* phage genomes form four apparent phylogenetic clusters (designated clusters 1 through 4 in the figure).

Phylogenetic analysis of the genomes of the 41 sequenced phages and all sequenced dairy streptococcal phages (with complete genomes) belonging to the *Moineauvirus* and *Vansinderenvirus* genera obtained from the NCBI RefSeq database highlights the limited genetic diversity of the *Vansinderenvirus* members irrespective of the factory of isolation (Italy or Austria) in this study. In contrast, the *Moineauvirus* isolates appear more genetically diverse with four apparent phylogenetic clusters (designated clusters 1 through 4, Figs 2 and S1, Table 2). Among the sequenced isolates from the current study, the majority (*n*=21) fall within *Moineauvirus* cluster 2, five fall within cluster 1 and eight within cluster 3. Interestingly, the cluster 4 *Moineauvirus* members vB_SthS_VA460 and vB_SthS_VA214 (whose genomes were derived from the NCBI repository) appear most closely related to the *Vansinderenvirus* members (Fig. 2).

**Fig. 3.**
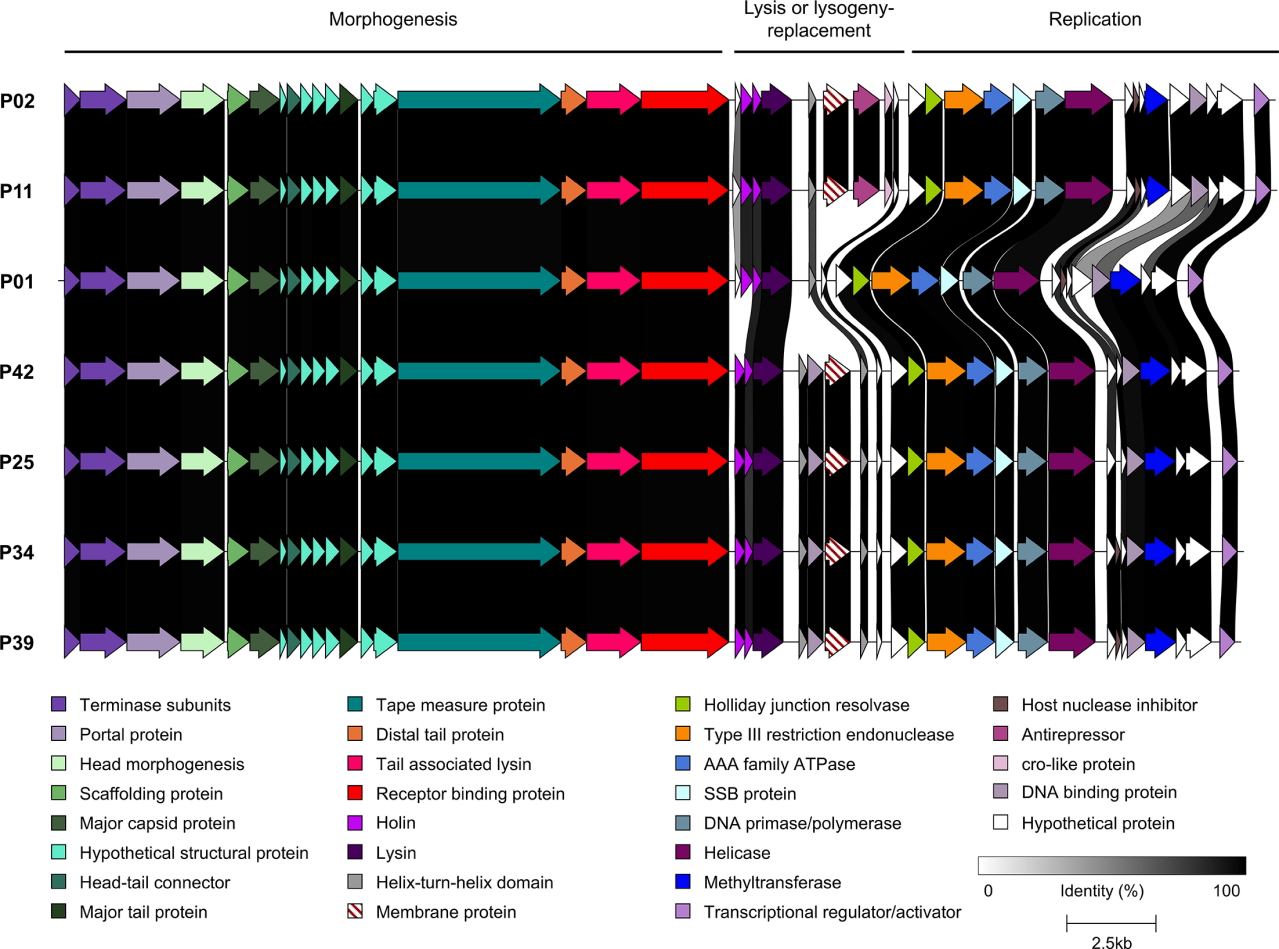
Genomic comparisons of the seven sequenced vansinderenviruses isolated within this study. The predicted genes of each genome are represented by arrows, which are colour-coded based on the (predicted) functions of the encoded proteins. These functions are indicated in the panel at the bottom of the figure. Percent amino acid identity shared between the encoded proteins is indicated by alignments ranging from grey to black (as indicated in the figure legend).

These findings prompted a detailed genome comparison of representative isolates of the individual phage clusters identified in the phylogenetic analysis. As mentioned above, limited genetic diversity was observed among the seven *Vansinderenvirus* members isolated in this study (Fig. 3). The genetic region encoding the morphogenesis functions (capsid and tail structural components and packaging-associated proteins) was almost identical among the genomes of all seven isolates. Within the replication-associated genomic region of their genomes, minor insertion/deletion events were observed. Interestingly, the variable gene content, such as the methyltransferase-encoding genes (Table S4), may reflect their adaptive responses to host-encoded phage-resistance systems. Additionally, most of the isolates (all but P01) encode a putative membrane or lipoprotein within the so-called lysis/lysogeny replacement region of the genome encoding two transmembrane domains at the N-terminal end, discussed further below. The isolate from the Austrian factory whey sample (P34) is identical to P25, P39 and P42 from the Italian factory, indicating a host-specific lineage of phages rather than a factory-specific phage population of vansinderenviruses.

**Fig. 4.**
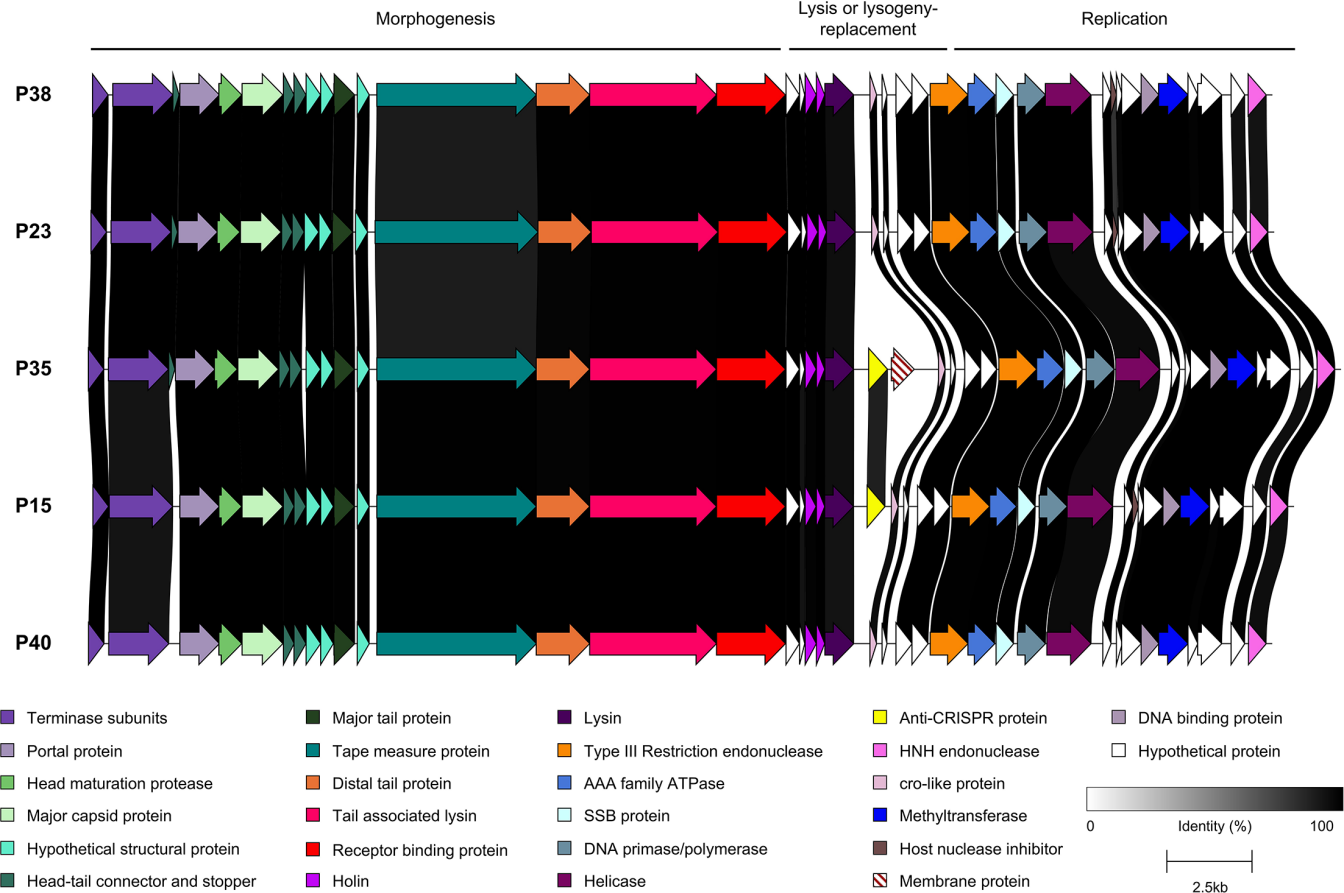
Genomic comparison of cluster 1 moineauviruses isolated within this study. The predicted functions of the proteins encoded by each gene are colour-coded (functions indicated in the panel located at the bottom of the figure). Percent amino acid identity shared between the encoded proteins is indicated by alignments ranging from grey to black (as indicated in the figure legend).

**Fig. 5.**
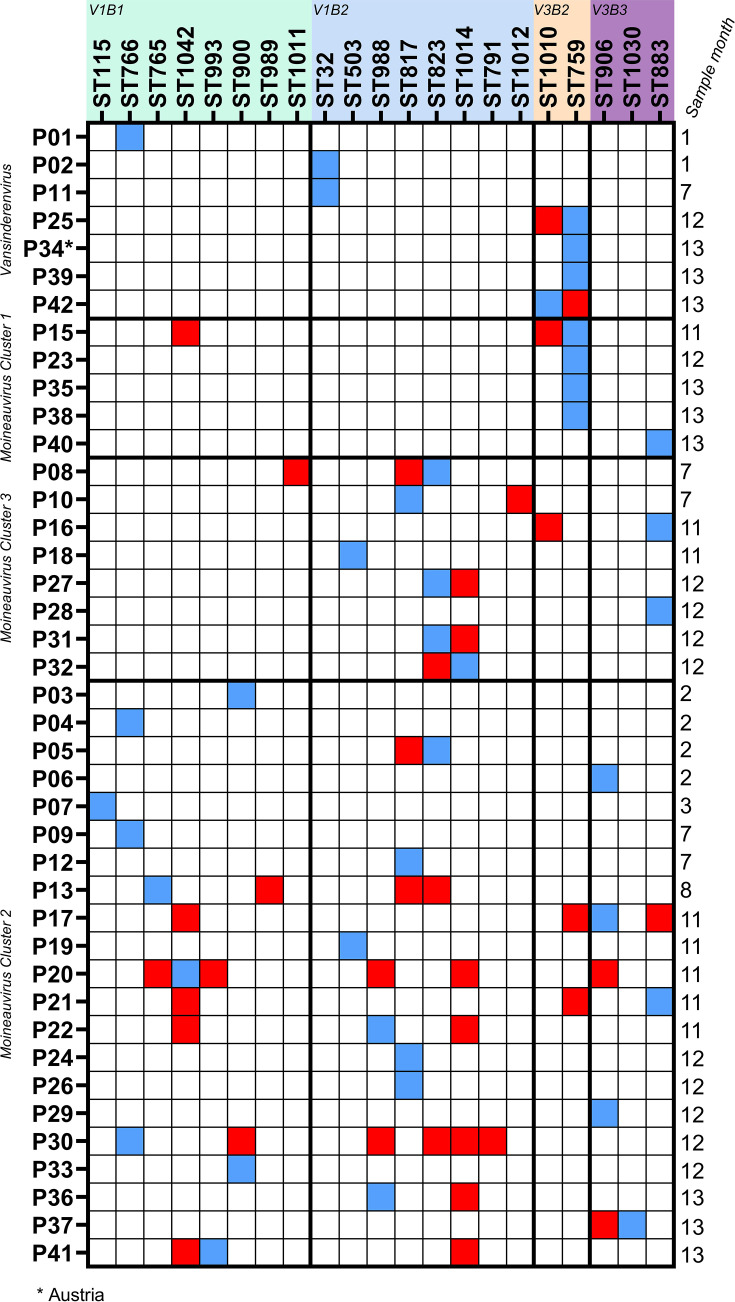
Host range of the 41 sequenced streptococcal phages isolated within this study. Host range was determined through spot assays against a panel of 58 *S*. *thermophilus* strains. The streptococcal phages are organized by their corresponding genera and clusters. The red squares indicate the primary host (i.e. host on which a particular phage was originally isolated), and blue squares indicate any additional host(s) a phage was capable of infecting. The sampling month at which a phage was isolated against a particular strain is shown at the right-hand side of the figure, and the phage genera and cluster (where applicable) of the phages are shown at the left. The sole phage (P34) isolated from the Austrian factory is indicated with an asterisk. White squares indicate no infection; only strains found to be sensitive to at least one phage have been included in the figure.

Within the five cluster 1 *Moineauvirus* phages isolated in this study, there is also very limited genome diversity, with the exception of apparent insertion/deletion events in the central region of their respective genomes. Within this genetic region, P15 and P35 encode predicted anti-CRISPR (Acr) protein-encoding genes (with 89.62% amino acid sequence identity to each other across 100% of the protein), while the P35 genome contains an additional gene encoding a membrane protein (unknown function) downstream of the predicted *acr* gene (Fig. 4). Notably, this encoded membrane protein is identical to the putative lipoprotein/membrane protein found in six of the *Vansinderenvirus* genomes (Fig. S2). Interestingly, all members of this *Moineauvirus* cluster possess genes that encode a predicted type III restriction endonuclease (indicative of a lysogenic ancestor) and a methyltransferase that is unique to cluster 1 *Moineauvirus* and the *Vansinderenvirus* isolate genomes (Figs 4 and S2). In order to determine if the infectivity of the isolated phages is limited to a single primary host (i.e. host on which a particular phage was originally isolated) of a particular *rgp* genotype, a host range analysis was performed against a panel of 58 strains (the 23 core strains plus an additional 35 *S*. *thermophilus* strains, Table S1) to determine any ‘secondary’ host(s) of the 41 phages (Fig. 5). The limited genetic diversity within this cluster corresponds well with the narrow host range observed for these phages (Fig. 5). Four of the five cluster 1 isolates from this study were shown to share the same primary host strain (P15, P23, P35, P38 all infect ST759), whereas P40 infects a different strain, ST883. Both of these host strains (ST759 and ST883) possess a V3 *rgp* variable genotype. Interestingly, P15 is also capable of infecting two additional strains (one which has a V1, the other a V3 *rgp* variable genotype). This broader host range of P15 may be attributed to the presence of an Acr-encoding gene on its genome, which is absent from the other cluster 1 members (which can only infect the primary host strain) besides P35, which also possesses a similar, yet distinct Acr-encoding gene (Fig. 5).

**Fig. 6.**
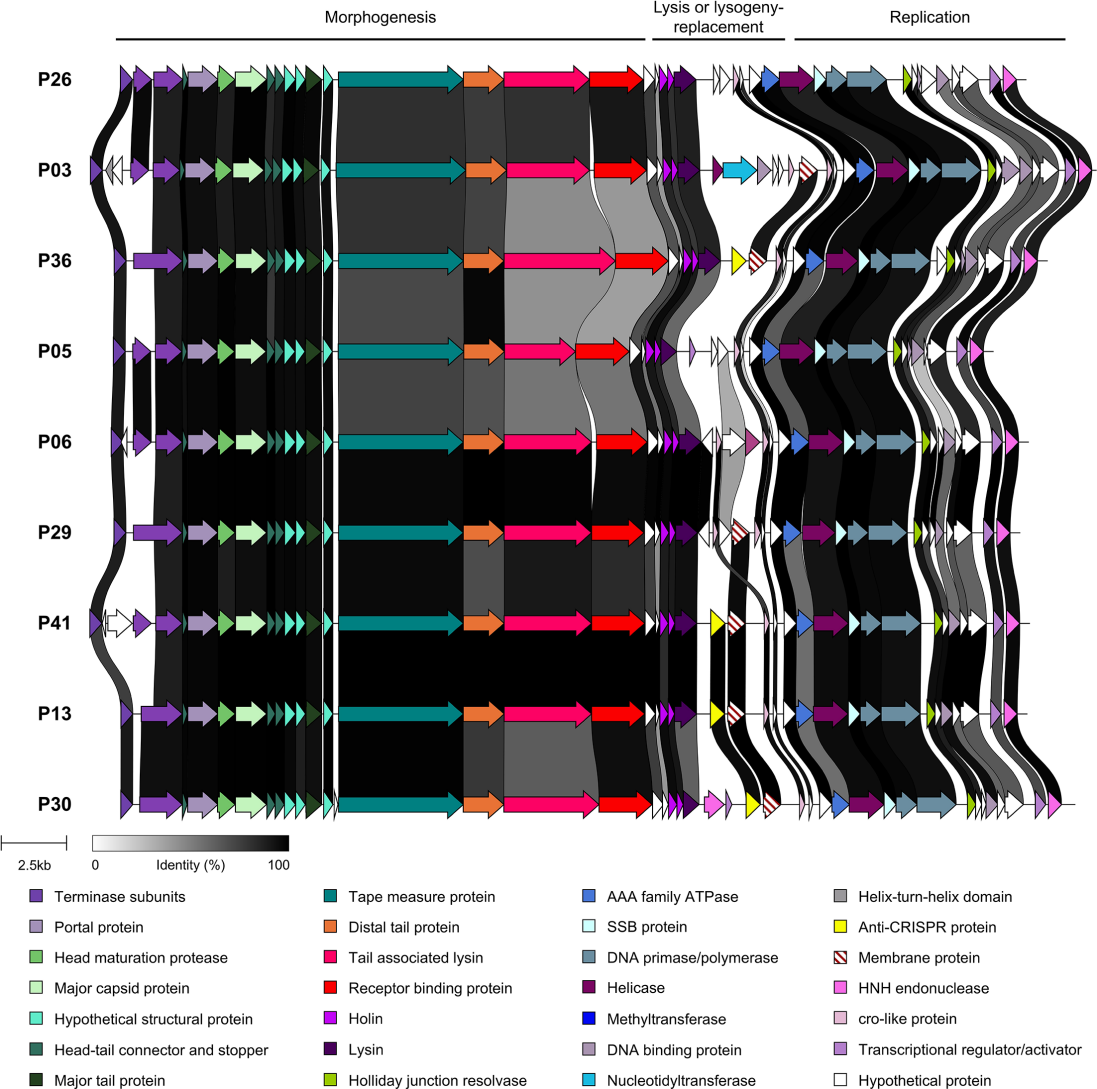
Genomic comparison of nine cluster 2 moineauviruses isolated from this study, representing the diversity observed within the cluster. The predicted functions of the proteins encoded by each gene are colour-coded (functions indicated in the panel located at the bottom of the figure). Percent amino acid identity shared between the encoded proteins is indicated by alignments ranging from grey to black (as indicated in the figure legend).

The *Moineauvirus* cluster 2, representing the most genetically diverse and populous *Moineauvirus* cluster, includes 64 phages, 21 of which were sequenced in this study. This genomic diversity is consistent with the higher number of primary host strains (of different *rgp* types) and unique host range profiles of these phages (Figs 5 and 6). Notably, within the replication regions of their genomes, only P19 encodes a distinct methyltransferase from those specified by other sequenced phages (Table S4). Similar to cluster 1 phages, it is the central region of the genome that appears to be the ‘hotspot’ of insertion and deletion events. Within this region, the P04, P07, P09, P13, P20, P22, P30, P36 and P41 genomes all share an identical insertion of an Acr-encoding gene (identified as an AcrIIa6 anti-CRISPR system based on HHPred analysis) and an identical gene encoding a predicted membrane-associated protein of unknown function downstream. Additionally, the genome of phage P30 possesses a gene encoding a predicted HNH endonuclease, which is unique to this phage [67]. For the remaining phages of this cluster, the residual genes within this genetic hotspot are largely of unknown function and represent unique gene content for those phages. The diversity of this region may reflect host-specific adaptations, and its gene content may represent a reservoir of as yet undefined host adaptation or phage counter-defence systems that will be investigated in the future.

**Fig. 7.**
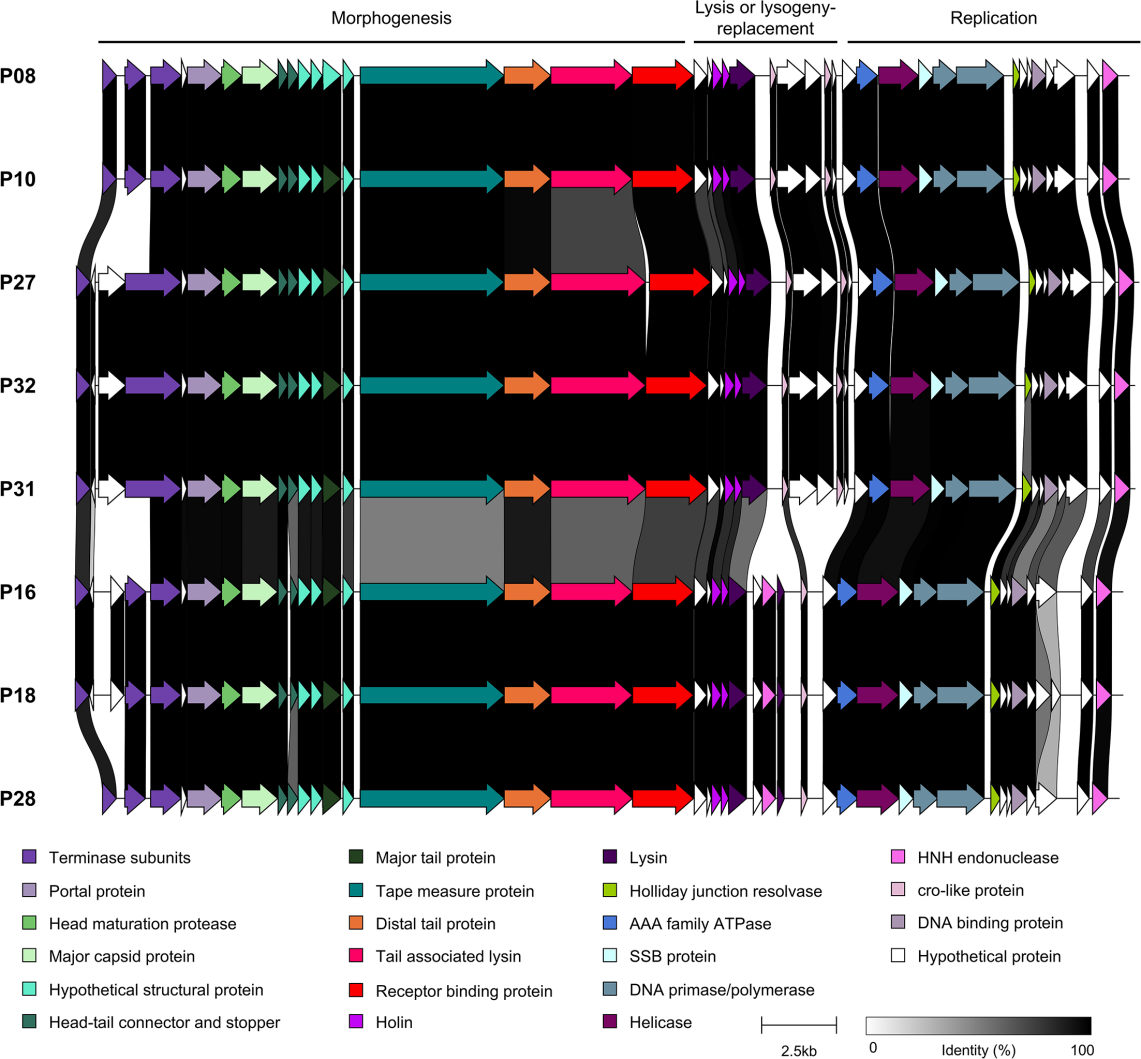
Genomic comparison of the cluster 3 moineauviruses sequenced within this study. The predicted functions of the proteins encoded by each gene is colour-coded (functions indicated in the panel located at the bottom of the figure). Percent amino acid identity shared between the encoded proteins is indicated by alignments ranging from grey to black (as indicated in the figure legend).

Cluster 3 moineauviruses isolated in the current study represent a group of eight phages (Fig. 7) which from a genetic perspective group together with a small number of previously sequenced streptococcal phage isolates (Fig. 2). These phages infect a limited number of host strains (Fig. 5), primarily those with a *rgp* V1 variable genotype (the exceptions being P16 and P28, which infect strains with a V3 genotype). In contrast to cluster 1 phage genomes analysed in this study, sequence divergence was observed within genes located in the structural gene module of these phages, most notably in those encoding tape measure protein, distal tail protein (Dit) and RBP. Additionally, the genomes of P08, P10, P27, P31 and P32 contain genes of unknown function within the central region of the genome that are not present in any of the other three phages of the cluster (P16, P18 and P28).

### Host-binding machinery and host range of isolated phages

#### Distal tail protein and tail-associated lysin

For the dairy streptococcal phage, the baseplate, located at the distal end of the phage tail, is the adhesion device involved in the recognition and binding of a phage to its host. The proteins involved in the formation of the baseplate are encoded by genes within the tail morphogenesis region and include the Dit, Tal and the RBP. The encoded adhesion devices of the sequenced *Vansinderenvirus* phages are well conserved, consisting of a ‘classical’ length (i.e. not possessing an additional insertion) Dit, comprising 239 residues, followed by a Tal of 508 residues. Consistent with a previous study regarding *Vansinderenvirus* Tal proteins, phage isolates from this study encode an N-terminal region containing a gp27-like domain followed by a previously functionally undefined fold [31].

In contrast, there is significantly more diversity among the *Moineauvirus* phages in terms of their host-binding machinery. Following the tape measure protein, the Dits of the sequenced moineauviruses are all ‘evolved’ [68] (~520 aa residues) and contain an insertion which, based on HHpred analysis, is similar to a CBM found in the evolved Dit of *Lacticaseibacillus casei* phage J-1 (5LY8), which is consistent with other reports of *Moineauvirus* Dits [31]. Despite the structural similarities between the *Moineauvirus* Dit proteins, there are notable differences in amino acid sequence composition, which may impact their corresponding binding specificity. The Dits of the sequenced moineauviruses within this study were, therefore, classified into one of three distinct Dit classes (1 – 3), based on amino acid similarity (Table 3).

**Table 3.** Characterization of the Dit, Tal and RBP encoded within the tail morphogenesis genetic module of the streptococcal phages sequenced within this study

Genera (cluster)	Phage	Dit		Tal		RBP	
		Size (aa)	Class	Size (aa)	Type	Size (aa)	Group
*Moineauvirus* (cluster 1)	P15	521	1	1,239	E	673	2
	P23	521	1	1,239	E	673	2
	P35	521	1	1,239	E	673	2
	P38	521	1	1,239	E	673	2
	P40	521	1	1,239	E	673	2
*Moineauvirus* (cluster 2)	P03	521	2	1,059	C	668	1
	P04	521	2	1,214	C	682	1
	P05	519	3	906	A	684	1
	P06	521	1	1,114	D	679	2
	P07	521	2	1,214	C	676	2
	P09	521	2	1,214	C	676	2
	P12	518	3	1,089	B	685	1
	P13	521	2	1,114	D	678	2
	P17	521	1	1,114	D	670	2
	P19	518	3	1,245	B	676	2
	P20	521	2	1,114	D	678	2
	P21	521	1	1,114	D	670	2
	P22	519	3	1,230	F	673	2
	P24	518	3	1,089	B	685	1
	P26	518	3	1,089	B	685	1
	P29	521	1	1,114	D	670	2
	P30	518	3	1,214	C	676	2
	P33	521	2	1,059	C	682	1
	P36	519	3	1,421	G	673	2
	P37	521	1	1,114	D	670	2
	P41	521	2	1,114	D	678	2
*Moineauvirus* (cluster 3)	P08	519	3	906	A	682	1
	P10	519	3	906	A	684	1
	P16	519	3	907	A	680	1
	P18	519	3	907	A	680	1
	P27	519	3	1,056	C	684	1
	P28	519	3	907	A	680	1
	P31	519	3	1,056	C	684	1
	P32	519	3	1,056	C	684	1
*Vansinderenvirus*	P01	239	Classical	508	–	821	–
	P02	239	Classical	508	–	821	–
	P11	239	Classical	508	–	821	–
	P25	239	Classical	508	–	818	–
	P34	239	Classical	508	–	821	–
	P39	239	Classical	508	–	821	–
	P42	239	Classical	508	–	821	–

Conversely, the Tal proteins encoded by the sequenced *Moineauvirus* genomes are much more structurally complex and diverse in size, ranging from 906 to 1,421 aa residues in length. The N-terminal domain (~400 residues) of the *Moineauvirus* Tals remains conserved with a gp27-like structural domain, consistent with previous studies [31,69]. Following the gp27-like domain, an Ig-like domain is present, which is in turn followed by extensions encoding one or more carbohydrate binding modules (CBMs) separated by collagen-like linker(s) of various lengths and 3*β* domain(s) [69]. Within the Tal extensions, there are notable differences among the analysed phages. Therefore, to further analyse the diversity of the Tals of the sequenced moineauviruses, the number and types of CBMs were identified. A previous study by Goulet *et al*. characterized and classified four CBMs (CBM_1 through to CBM_4) based on structural analysis of the Tals of five streptococcal phages (belonging to the *Moineauvirus* and *Brussowvirus* genera) [69]. Within the Tals of the isolated moineauviruses, the CBMs CBM_2, CBM_3 and CBM_4 could be identified (Table S5, Fig. S3). Additionally, a number of Tals were found to contain a novel CBM domain, hereafter named CBM_5 (Table S5, Fig. S3), which was identified by Dali as a glycoside hydrolase family protein that contains a CBM family 35 domain at the C-terminal end. Additionally, three unknown domains hereafter called UNK_6, UNK_7 and UNK_8 were identified in several phages, yet did not share high identity (*Z* score <10) with other previously described structures available on Dali (Tables S5 and S6, Fig. S3). Following the identification of the CBMs and unknown domains encoded within the Tal of the 35 moineauviruses, the Tals were classified into seven different types (A–G) based on the combinations of CBMs or unknown domains (Tables 3 and S5). Tals of the same type may vary based on size due to the length or number of collagen-like linker regions and 3*β* domains.

#### Receptor-binding protein

**Fig. 8.**
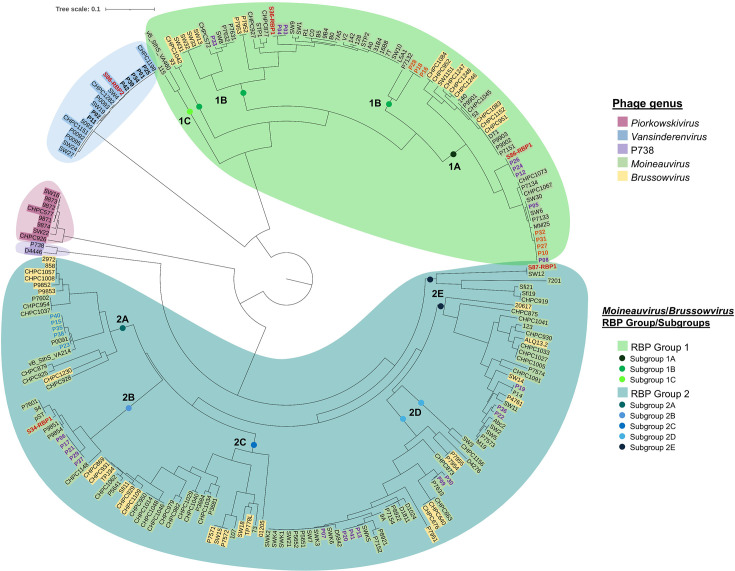
Phylogenetic trees of the RBP head domains of the 41 streptococcal phages isolated in this study (bolded text) alongside those of publicly available RBP head domains (183) retrieved from phages belonging to all streptococcal genera. The *Moineauvirus* members sequenced within this study are coloured according to which genetic cluster they belong to: blue for cluster 1, purple for cluster 2 and orange for cluster 3 (as illustrated in the figure legend). RBPs retrieved from phageome assemblies are in bold and red. The identified RBP groups and subgroups are colour-coded according to the figure legend.

The ORF following the Dit and Tal has previously been determined to represent the bona fide RBP-encoding gene involved in the specific recognition and binding of a host saccharidic receptor [31]. To gain a better understanding of the diversity of RBPs among streptococcal phages, a phylogenetic tree of the RBP head domains of 183 publicly available *S. thermophilus* phages (from all 5 genera), as well as the 41 novel moineauviruses and vansinderenviruses sequenced within this study, was generated (Fig. 8). The RBP head domains of the *Piorkowskivirus, Vansinderenvirus* and P738 phages all cluster within their distinct genera and share high amino acid identity at the genus level (including the vansinderenviruses described within this study). However, interestingly, the *Moineauvirus* and *Brussowvirus* RBP head domains cluster together (rather than strictly among their own genera), forming two apparent *Moineauvirus/Brussowvirus* RBP groups (1 and 2) based on amino acid similarities within the head domain (a minimum of 50% aa identity across the RBP head domain is shared among group members). Among the moineauviruses sequenced within this study, the majority (*n*=19) possess a group 2 RBP and the remaining 15 a group 1 RBP. Also notably, *Moineauvirus/Brussowvirus* members possessing a group 2 RBP share higher aa similarity to the RBP head domain of P738 phages than those of *Moineauvirus/Brussowvirus* RBP group 1.

### Phageome analysis

**Fig. 9.**
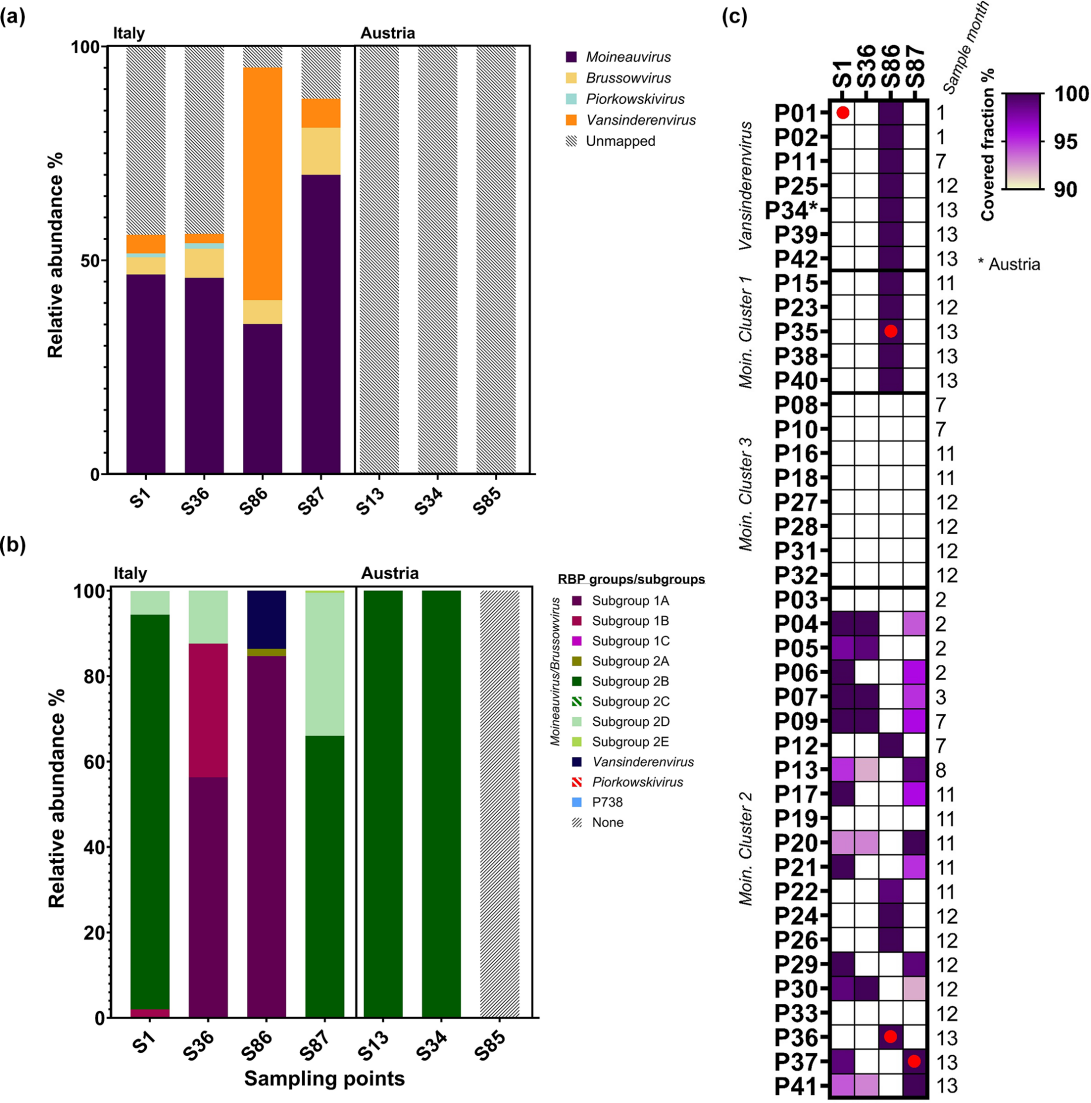
Phageome analysis dissecting the phage diversity present in seven whey virome samples collected from an Italian and Austrian dairy fermentation factory during the early, mid and late sampling points. (**a**) Relative abundance of streptococcal phage genera (*Moineauvirus*, *Brussowvirus, Piorkowskivirus* and *Vansinderenvirus*). (**b**) Relative abundance of streptococcal phage RBP groups/subgroups. Unmapped reads are depicted as diagonally striped, grey lines. (a and b) Whey samples (S#) are named according to sampling times, representing the early (S1, S13), mid (S34, S36) and late (S85–S87) sampling points. (**c**) Presence or absence of an identical or closely related (>85% identity, >95% coverage) *Moineauvirus* or *Vansinderenvirus* phage genomes in the whey virome, determined through reads mapping of the phage genomes against the raw whey virome reads (in order to calculate the covered fraction). The minimum covered fraction represented in the figure is 90%, with the squares being coloured according to the calculated covered fraction (between 90 and 100%). The samples from the Austrian (S13, S34, and S85) factory are excluded from this figure as none of the phages were detected in the samples (using the defined parameters). A red circle indicates where a phage was isolated from a particular whey sample.

While phage screening and isolation processes are crucial to identifying dominant phages and unique phage–host combinations, the range of host strains applied may bias the screening process. Therefore, to evaluate if and to what extent the isolated phages in this study reflect the overall phageome (assembled phage-like genetic components) present during the fermentations, the viromes of seven whey samples (four from the Italian factory and three from the Austrian) were extracted and sequenced (Table 4). Whey samples were chosen from an early (months 1–2), mid (month 6) and late (month 12/13) sampling points for both the Italian and Austrian factories (Table 4). An average DNA sequencing output of 3,986,451 high-quality reads (after quality filtering) per sample was generated. Initial taxonomic classification of the assembled phageome-derived phage contigs established that dairy streptococcal and lactococcal phages (albeit to a lesser extent) were determined to be by far the most prevalent and relevant phage species in the whey viromes. Therefore, reads mapping was performed against a database of all publicly available phage dairy * L. lactis/cremoris* and *S. thermophilus* reference phage sequences (retrieved from NCBI) in order to further establish the prevalence and diversity of phages in the samples. No reads aligned to any of the reference genomes in any of the three Austrian samples. These unmapped reads may correspond to contaminating bacterial DNA or unique phage reads that are distinct from those currently available on NCBI. However, the samples from the Italian factory generated reads of which between 56 and 88% mapped to *S. thermophilus* phage genera(s). Moineauviruses were shown to be the dominant phages in whey samples S1, S36 and S87 derived from the Italian factory. Conversely, vansinderenviruses were the dominant genus in S86, followed by moineauviruses (Fig. 9a). Interestingly, samples S86 and S87 were collected at the same sampling point, though they were collected from different vats utilizing different starter cultures (at the time of collection). From the culture-based phage screening, the *Vansinderenvirus* P01 was isolated from sample S1, while the *Moineauvirus* isolates P35 and P36 were isolated from sample S86, and the *Moineauvirus* P37 was isolated from sample S87. These isolates do, therefore, not necessarily reflect the dominant/most abundant phages in the samples, as based on phageome analysis, yet seem to represent rather host-specific isolates that can propagate under laboratory conditions.

**Table 4.** Sample information for the sequenced whey viromes

Sample	Factory	Date of collection	High-quality filtered reads (bp)	% mapped reads*	BioSample accession no.
S1	Italy	Oct. 2022	5,169,622	56.0%	SAMN53757943
S13	Austria	Nov. 2022	3,194,291	0.0%	SAMN53757944
S34	Austria	Mar. 2023	4,637,475	0.0%	SAMN53757945
S36	Italy	Mar. 2023	7,088,286	56.2%	SAMN53757946
S85	Austria	Oct. 2023	293,347	0.0%	SAMN53757947
S86	Italy	Oct. 2023	5,630,428	95.2%	SAMN53757948
S87	Italy	Oct. 2023	2,071,982	88.0%	SAMN53757949

*Reads mapped against the database of publicly available dairy streptococcal and lactococcal phages.

Additionally, reads were identified that mapped to *Brussowvirus* sequences in all three whey viromes from the Italian factory; however, this may be due to the genetic similarities that have been observed between moineauviruses and brussowviruses (within both the tail morphogenesis and replication regions) or indicate that these phages may be present in the dairy facility, however, in lower abundance. The same is true for piorkowskiviruses, which were identified in low abundance in the whey viromes S1 and S36 (Fig. 9a). While the Austrian factory samples did not reveal any reads that map against any phage sequence in the employed database, a single *Vansinderenvirus*, P34, was nonetheless isolated from sample S85.

To gain further insights into the diversity of streptococcal phages within the samples, additional reads mapping was performed using representative RBP head domain sequences corresponding to the newly defined *S. thermophilus* phage RBP groups/subgroups. In the Italian factory samples S1 and S87, subgroup 2B RBPs were the most dominant (at 92% and 66%, respectively). However, subgroup 1A was the most dominant in S36 (56%) and S86 (85%). Additionally, S86 was the only sample from the Italian factory where *Vansinderenvirus* RBPs were detected (14%). Interestingly, a *Vansinderenvirus* member was isolated from S1, indicating that vansinderenviruses were present in the Italian factory since the first sampling point, most likely in low abundance (at least in that particular vat). In the Austrian samples, despite no reads aligning to any of the streptococcal reference genomes, there were still reads corresponding to the *Moineauvirus/Brussowvirus* RBP subgroup 2B (in both S13 and S34 but not in S85).

Attempts were made to identify complete or nearly complete (i.e. >25 Kbp) streptococcal phage genomes from the assembled phageomes (assembled using both METAnnotatorX2 and Phables). However, only the phageomes of S34 (Austria) included a streptococcal phage contig larger than 25 Kbp; the streptococcal phage contigs in the remaining samples were significantly below this cut-off (Table S7). S34-Contig_1 was 28,139 bp in length and found to be most similar to *Moineauvirus* 94 (GenBank accession number: MH375594; QC 83%, PI 95.33%). Notably, S34-Contig_1 is not identical to the sequenced moineauviruses found in the primary Italian factory; however, it displays similarity to *Moineauvirus* cluster 2 member P06 (sharing 96.54% identity across 84% of the genome). The phageome contigs (irrespective of length) were additionally manually scrutinized for the presence of RBP-encoding sequences. Among the Austrian samples, only one distinct *Moineauvirus* RBP-encoding gene was identified in S34, belonging to subgroup 2B, consistent with the reads mapping results (Fig. 9b). For the Italian factory samples, two distinct RBPs were found in S86, one clustering among the vansinderenviruses and the other being a *Moineauvirus* RBP belonging to subgroup 1A (Fig. 9b). The remaining two *Moineauvirus* RBPs were found in S36 (subgroup 1B) and S87 (subgroup 2E).

The isolated streptococcal phage genomes were mapped against the virome reads to determine which may be persistent or resident phages in the fermentation facilities (Fig. 9c). In the Italian factory, the vansinderenviruses and *Moineauvirus* cluster 1 phages were only detected in S86 at the late sampling point. Additionally, *Moineauvirus* cluster 2 phages P12, P22, P24, P25 and P36 were only present in S86 (and not at the other sampling points), with P36 being isolated from this sample. Many of the *Moineauvirus* cluster 2 members were identified in at least one of the other three samples (S1, S36 and/or S87), with several phages (or at least closely related phages) persisting across all three sample periods (P04, P07, P09, P13, P20, P30 and P41). Interestingly, four *Moineauvirus* cluster 2 phages (P03 and P33) and all *Moineauvirus* cluster 3 members were not detected in any of the sequenced whey viromes, indicating fermentation-specific populations distinct from those of the phageome-analysed samples (Fig. 9c). There was no significant coverage for any of the sequenced phage genomes in the Austrian samples.

## Discussion

In recent decades, dairy fermentations in Europe have increased in scale and intensity, partly due to the abolition of the milk quota in the European Union in 2015. This increased intensity of production has placed significant pressure on the fermentations and the ability of producers to ensure the best hygienic practices. Phages that are resident or prevalent in individual factories are maintained by the repeated application of the same or similar starter cultures. Since fermentation facilities and processes are not sterile, it is impossible to eradicate phages. However, knowledge of the resident and/or prevalent phages in a given factory is helpful to define factory-specific solutions to this challenge. Furthermore, to strategically plan for future production years, it is also essential to understand how transient blooms of individual phages may affect certain strains within the starter cultures used in their fermentations to provide a level of predictability and stability to production operations. Therefore, in the current study, cheese whey samples derived from two factories in Austria and Italy using the same starter culture systems were surveyed for the presence and diversity of phages across a 1-year period.

The Austrian factory whey samples appeared to contain a very limited number of phages against the panel of strains in our collection. This is a remarkable finding given that this factory employs starter cultures that incorporate these strains routinely during the testing period. It is plausible that the factory also applied other cultures in rotation to minimize the risk of phage proliferation. Furthermore, as the Austrian factory is more modern and performs larger-scale fermentations than the Italian factory, this factory likely exercised very strict sanitation practices, thereby reducing the phage load in the factory. Only a single *Vansinderenvirus* isolate was identified in this study from one of the 38 Austrian factory whey samples, highlighting the low abundance of phages present. Conversely, phages were isolated from 20 of the 47 whey samples derived from the Italian factory. In total, 40 phages were isolated from these 20 whey samples, and among them were 6 *Vansinderenvirus* and 34 *Moineauvirus* isolates. The dominance of *Moineauvirus* phages is consistent with previous reports [12,22, 24, 29, 37]. It is also evident that *Vansinderenvirus* members are consistently being isolated in recent studies, and this observation is likely associated with the application of particular strains in fermentations that enrich for these phages. This hypothesis is supported by the apparent narrow host range of the isolated *Vansinderenvirus* members in this study. Overall, there appears to be very limited genetic diversity among *Vansinderenvirus* members based on phylogenetic analysis of all sequenced isolates of the genus, while their receptor-binding proteins are also highly similar. This suggests that there is limited pressure to evolve and adapt to alternative hosts, possibly due to the consistent presence of at least one strain with a suitable receptor moiety within starter cultures used routinely in industry. While the saccharidic receptor for members of the *Brussowvirus*, *Piorkowskivirus* and P738 phages has been identified, the receptor moiety of *Vansinderenvirus* members remains unknown. However, in the case of both moineauviruses and vansinderenviruses, it has been proposed that EPS is the receptor [26,33]. RGP acts as a receptor for two of the three genera (*Brussowvirus* and P738 phages) for which the receptor is known, and we recently established a rapid PCR-based typing tool for dairy streptococcal strains based on their *rgp* genotype [30,34, 35]. Based on *rgp* genotypic data, the host strains of the *Vansinderenvirus* isolates from the present study possess different *rgp* genotypes (V1B1, V1B2 and V3B2 genotypes). This indicates that RGP is not the primary or sole determinant of host specificity for members of this phage genus. Further studies will be required to establish the specific nature of the receptor; however, it seems plausible that, like the *Piorkowskivirus* members, the exopolysaccharide acts as the receptor for these phages. Exopolysaccharides produced by *S. thermophilus* and the gene clusters associated with their biosynthesis are known to be diverse and would explain the narrow host range of these phages [36,37, 70, 71]. However, it is also possible that there is a two-step adsorption process for these phages similar to lactococcal *Ceduovirus* members, which first reversibly bind to a saccharidic receptor and then irreversibly bind to the phage infection protein or its analogue YjaE [72,73].

Phylogenetic analysis of the sequences of the isolates from this study revealed that while members of the *Vansinderenvirus* genus all cluster closely together with genus members from other studies and available in public databases, there is considerable diversity among the *Moineauvirus* members, with four *Moineauvirus* clusters observed. This overall genetic diversity is mirrored and further complicated by an apparent sharing of receptor-binding proteins between members not only of *Moineauvirus* phage clusters (based on overall gene content) but also between *Moineauvirus* and *Brussowvirus* members. These two genera of phages have dominated in phage screening surveys over the past number of decades and have likely co-existed in the dairy niche creating opportunities for recombination events to occur. Since the receptor for only one *Brussowvirus* (SW13, RBP Group 1B) is currently defined (and is an RGP moiety), it is not possible to conclusively identify the receptor moiety for all members of the *Moineauvirus/Brussowvirus* members based on RBP phylogeny alone. While binding domains encoded by other proteins present in the phage baseplate (such as the Tal and Dit) may play a role in host recognition, this hypothesis would require further experimental validation. While the presence of multiple carbohydrate-binding domains in the adhesion device-associated proteins of these phages makes it tempting to speculate that the receptor is most likely a saccharide, alternative binding strategies remain a possibility and will require further investigation in future studies. The identification of the receptor for this key genus of phages represents a key area of future development to fully unravel the complexity of phage–host interactions in *S. thermophilus*.

By mapping the reads from virome sequence data from the early, mid and late sampling months onto the genome sequences of the isolated phages, we investigated if certain phages are prevalent across the sampling period in the Italian factory. This analysis revealed that cluster 2 *Moineauvirus* members represent the most prevalent phages for this factory across the sampling period. The phages within *Moineauvirus* cluster 2 are also the most genetically diverse and abundant in public databases. However, a notable omission from this analysis was the apparent lack of a phage in sample S1 with at least 90% coverage of phage P01 (as this phage was isolated from sample S1). This suggests that phage P01 was present in relatively low abundance and that the phage screening allowed its specific enrichment and isolation. It may also reflect the limitations of the DNA extraction procedures and highlights the need for ongoing refinement and improvement of these procedures from food samples, where yields are often low [74].

In the present study, it was aimed to compare the phages present in two factories from distinct geographical locations using the same starter culture systems routinely (likely in addition to other cultures used for other factory-specific products). Additionally, the factories differed in fermentation practices. Where the Austrian factory performed more large-scale and automated fermentations, the Italian factory was much smaller and less modern in comparison and, therefore, would run many fermentations per day without cleaning the vats in between fermentations. These differences in fermentation practice likely contributed to the difference in phage populations of the two factories, with the Austrian factory samples having both very limited phageome-derived phage sequences and only one isolated phage. This finding suggests that local practices in terms of production intensity and starter culture rotation, as well as hygiene practices, dictate the abundance of phages in the fermentations and can either facilitate or limit phage proliferation (and possible recombination events). Despite the higher abundance of phages in the Italian factory, it is important to note that the acidification process of the fermentation was not inhibited, thereby still allowing for the consistent production of the desired cheese product. The abundance and diversity of phages in the Italian factory suggest that the particular starter cultures analysed in this study are applied frequently and possibly at high intensity in comparison to the Austrian factory. Such information is essential for starter culture providers to inform and support dairy fermentation factories in the selection of starter cultures to reduce the risk of phage proliferation and the associated impacts on the final product quality and consistency. Furthermore, the observed increase in the number of strains infected by phages in the whey samples across the 13-month period is partially explained by the adaptation of certain phages to BIMs applied in these cultures. However, it is also likely that the diversity of phages in the Italian plant may facilitate recombination events that underpin the evolution of these phages, allowing them not only to circumvent the BIMs but also the phage resistance systems of additional (and previously phage-resistant) strains. The dynamics of the starter culture population may also vary in different fermentation facilities and environments, creating unique profiles of ‘dominant’ strains that may become the target of obligately circumvent phages and lead to distinct phage population dynamics in different production facilities and geographical locations. Therefore, it is essential to continue to understand the ecology and dynamics of phage populations in global dairy fermentation facilities to define the key factors that underpin the success, evolution and adaptation of phages in dairy fermentations.

## Conclusion

This study highlights the benefits of combining culture-based and culture-independent approaches to define phage populations in specific fermentations and/or factories. However, it also underlines the current limitations of phageome-based studies for the assembly of genomes of closely related phages, which is a common scenario in modern industrial food fermentations, particularly where defined starter cultures are applied. Furthermore, this study revealed that *Brussowvirus* and *Moineauvirus* receptor-binding proteins are not confined within the boundaries of a specific phage genus. The identification of several groups of their receptor-binding proteins based on phylogenetic analysis creates a roadmap to begin to unravel the complex interactions of dairy streptococcal phages and their hosts.

## Supplementary material

10.1099/mgen.0.001729 Supplementary Material 1.

## Abbreviations

ANI: average nucleotide identity
BIM: bacteriophage-insensitive mutants
CBMs: carbohydrate binding modules
CRISPR: clustered regularly interspaced short palindromic repeats
Dit: distal tail protein
EPS: exopolysaccharide
NCBI: National Center for Biotechnology Information
RBP: receptor-binding protein
RGP: rhamnose-glucose polysaccharide
R/M: restriction/modification
Tal: tail-associated lysin
