# Supplementary material for: Diversity and prevalence of dairy streptococcal phages in two European dairy fermentation facilities over a one-year period
Source: Microb Genom. 2026 May 26;12(5):001729. doi: 10.1099/mgen.0.001729 (PMC13207995; doi:10.1099/mgen.0.001729)
Supplement: Supplementary Material 1. [file mgen-12-01729-s001.pdf]

Supplementary Table S1. List of 58 *S. thermophilus* strains used in this study and their *rgp* genotype.

***S. thermophilus* strain**	***rgp* type†**
ST11*	V1B2
ST14	V4B1
ST21	V3B3
ST32	V1B2
ST47	V2B2
ST51	V3B3
ST74	V3B3
ST75	VxB2
ST76	UND
ST115	V1B1
ST117	UND
ST318	VxB1
ST386	V3B3
ST433	V1Bx
ST457	VxB2
ST460	V3B3
ST473	V3B3
ST495	V1B2
ST503*	V1B2
ST504	V3B3
ST505	V3B3
ST511	V3B3
ST581	VxB1
ST614*	V3B3
ST652	UND
ST671	V3B3
ST675	V3B3
ST684	V4B1
ST759*	V3B2
ST765*	V1B1
ST766*	V1B1
ST772*	V3B3
ST791*	V1B2
ST817*	V1B2
ST823*	V1B2
ST883*	V3B3
ST896*	V3B3
ST900*	V1B1
ST906*	V3B3
ST917	V3B3
ST945*	V1B2
ST976	V3B3
ST980*	V1B2
ST986	V1Bx
ST987	V1Bx
ST988* (BIM of ST32)	V1B2
ST989	V1B1
ST990	V1B1
ST991	VxB1
ST992	V1B1
ST993* (BIM of ST115)	V1B1

ST1010* (BIM of ST759)	V3B2
ST1011	V1B1
ST1012	V1B2
ST1014* (BIM of ST823)	V1B2
ST1018* (BIM of ST772)	V3B3
ST1030* (BIM of ST906)	V3B3
ST1042* (BIM of ST115)	V1B1

*Indicates 23 core strains used in initial phage screening panel.
†UND; could not be determined using colony PCR.

Supplementary Table S2. Sample information for the 85 cheese whey samples collected from the Italian and Austrian dairy facilities between October 2022 and October 2023.

Whey sample	Dairy facility	Collection date
S1*	Italy	Oct. 2022
S2	Italy	Oct. 2022
S3	Italy	Oct. 2022
S4	Italy	Oct. 2022
S5	Austria	Oct. 2022
S6	Austria	Oct. 2022
S7	Austria	Oct. 2022
S9	Italy	Nov. 2022
S10	Italy	Nov. 2022
S11	Italy	Nov. 2022
S12	Italy	Nov. 2022
S13*	Austria	Nov. 2022
S14	Austria	Nov. 2022
S15	Austria	Nov. 2022
S16	Italy	Dec. 2022
S17	Italy	Dec. 2022
S18	Austria	Dec. 2022
S19	Austria	Dec. 2022
S20	Austria	Jan. 2023
S21	Austria	Jan. 2023
S22	Austria	Jan. 2023
S23	Italy	Jan. 2023
S24	Italy	Jan. 2023
S25	Austria	Feb. 2023
S26	Austria	Feb. 2023
S27	Austria	Feb. 2023
S28	Italy	Feb. 2023
S29	Italy	Feb. 2023
S30	Italy	Feb. 2023
S31	Italy	Feb. 2023
S32	Austria	Mar. 2023
S33	Austria	Mar. 2023
S34*	Austria	Mar. 2023
S35	Italy	Mar. 2023
S36*	Italy	Mar. 2023
S37	Italy	Mar. 2023
S38	Austria	Apr. 2023
S39	Austria	Apr. 2023
S40	Austria	Apr. 2023
S41	Italy	Apr. 2023
S42	Italy	Apr. 2023
S43	Italy	Apr. 2023
S44	Italy	Apr. 2023
S45	Austria	May 2023
S46	Austria	May 2023
S47	Austria	May 2023
S48	Italy	May 2023
S49	Italy	May 2023
S50	Italy	May 2023

S51	Italy	May 2023
S55	Italy	Jun 2023
S56	Italy	Jun 2023
S57	Italy	Jun 2023
S58	Italy	Jun 2023
S59	Austria	Jun 2023
S60	Austria	Jun 2023
S61	Austria	Jun 2023
S62	Italy	July 2023
S63	Italy	July 2023
S64	Italy	July 2023
S65	Italy	July 2023
S66	Austria	July 2023
S67	Austria	July 2023
S68	Austria	July 2023
S69	Italy	Aug. 2023
S70	Italy	Aug. 2023
S71	Italy	Aug. 2023
S72	Italy	Aug. 2023
S73	Austria	Aug. 2023
S74	Austria	Aug. 2023
S75	Austria	Aug. 2023
S76	Austria	Sept. 2023
S77	Austria	Sept. 2023
S78	Austria	Sept. 2023
S79	Italy	Sept. 2023
S80	Italy	Sept. 2023
S81	Italy	Sept. 2023
S82	Italy	Sept. 2023
S83	Austria	Oct. 2023
S84	Austria	Oct. 2023
S85*	Austria	Oct. 2023
S86*	Italy	Oct. 2023
S87*	Italy	Oct. 2023
S88	Italy	Oct. 2023
S89	Italy	Oct. 2023

*Wheys selected for virome sequencing.

Supplementary Table S3. Sensitive host strains, number of phage isolates, and sampling months from which phages were isolated. *S. thermophilus* strains part of the core strain panel are indicated by an asterisk.

Propagating host	No. phage isolates	Sampling month of isolation
**ST759***	12	Month 2, 4, 11, 12, 13
**ST766***	12	Month 1, 2, 5, 6, 7, 12
**ST823***	11	Month 1, 2, 6, 7, 12
**ST906***	10	Month 2, 3, 5, 9, 12, 13
**ST883***	7	Month 8, 11, 12, 13
**ST791***	5	Month 2, 6, 7, 12
**ST900***	5	Month 6, 7, 12
**ST503***	2	Month 8, 11
**ST765***	3	Month 3, 4, 5
**ST817***	3	Month 4, 7, 12
**ST988***	3	Month 11, 12, 13
**ST993***	3	Month 11, 13
**ST1011**	2	Month 7
**ST1012**	2	Month 7
**ST1030***	2	Month 13
**ST32**	2	Month 1, 7
**ST989**	2	Month 8
**ST1010***	1	Month 13
**ST1014***	1	Month 12
**ST115**	1	Month 3
**ST917**	1	Month 8
**ST976**	1	Month 8
**ST986**	1	Month 9
**ST987**	1	Month 9
**ST990**	1	Month 9
**ST991**	1	Month 9
**ST992**	1	Month 9
**ST1042***	1	Month 11

Supplementary Table S4. Analysis of methyltransferases (MTases) encoded with the sequences *S. thermophilus* phage genomes using REBASE.

Mtases ORFs	Length	Math to	Type	Meth	Identity (%)
P40_34, P38_36, P35_37, P15_36, P42_37, P39_38, P25_37, P01_37, P23_36, P34_38	287	M.Sph5093ORFAP	2	C5	68%
P02_38, P11_38	214	M.Lrh6ORF13185P	1	N6A	46%
P19_31	250	M.SthPhi7201ORFAP	2	-	99%

Supplementary Table S5. Analysis of the CBMs or unknown domains (UNK) identified within the variable regions (VR1 and VR2) Tals of the sequenced Moineauviruses using AlphaFold3 and the Dali server. CBM_2, CBM_3, and CBM_4 had been previously defined [1]. Unknown domains did not share high identity (Z score <10) to other previously described structures on Dali.

Type	Phages*	Size range (aa)	VR1	VR2
A	P05, **P08**, P10, P16, P18, P28	906-907	-	CBM_4 (2zey; 15.0)
B	P12, **P19**, P24, P26	1089-1249	CBM_2 (5e7t; 29.4)	CBM_4 (2zey; 15.0)
C	P03, P04, P07, P09, P30, P33, P27, **P31**, P32	1056-1214	CBM_5 (5x7p; 12.9)	CBM_4 (2zey; 14.6)
D	P06, P13, **P17**, P20, P20, P21, P29, P37, P41	1114	CBM_5 (5x7p; 12.6)	CBM_3 (3p6b; 12.2)
E	**P15**, P23, P35, P38, P40	1239	UNK_6	CBM_4 (2zey; 14.8)
F	**P22**	1230	UNK_7	CBM_4 (2zey; 14.6)
G	**P36**	1421	UNK_8	CBM_4 (2zey; 10.8)

*Representative phage used for structural analysis in **bold**.

Supplementary Table S6. The top 20 Dali hits for the three unknown domains (UNK_6, UNK_7, and UNK_8) identified in the Tals of three representative Moineauviruses (P15, P22, and P36).

**P15 UNK_6**							
No:	Chain	Z	rmsd	lali	nres	%id	PDB Description
1	6frg-A	5.9	3.3	58	158	14	MOLECULE: REPLICATIVE DNA HELICASE;
2	1mi8-A	5.8	2.3	54	141	13	MOLECULE: DNAB INTEIN;
3	6qaz-A	5.7	3.5	58	128	14	MOLECULE: CYANOPHAGE-LIKE GP41-1 INTEIN;
4	6rpp-A	5.7	4.2	61	168	11	MOLECULE: CELL DIVISION CONTROL PROTEIN;
5	5o9j-B	5.7	4	62	183	21	MOLECULE: TRANSCRIPTION INITIATION FACTOR IIB,TRANSCRIPTION
6	4o1r-A	5.7	3.4	58	142	12	MOLECULE: REPLICATIVE DNA HELICASE;
7	6riz-A	5.6	3.3	58	127	14	MOLECULE: GP41-1 INTEIN;
8	6rpq-A	5.5	4	63	249	17	MOLECULE: UBIQUITIN-LIKE PROTEIN SMT3,1108AA LONG HYPOTHETI
9	6fre-A	5.4	2.4	54	154	15	MOLECULE: REPLICATIVE DNA HELICASE,REPLICATIVE DNA HELICASE
10	6vgw-A	5.4	2.4	52	145	17	MOLECULE: VIDAL;
11	8ubs-B	5.4	3.1	59	150	8	MOLECULE: NRDJ-1;
12	6vgv-A	5.4	2.3	52	145	17	MOLECULE: VIDAL;
13	7qss-A	5.3	3.5	59	432	14	MOLECULE: V-TYPE ATP SYNTHASE ALPHA CHAIN;
14	4e2u-A	5.3	3.4	59	168	14	MOLECULE: PHO RADA INTEIN;
15	4o1s-A	5.2	3.6	61	170	21	MOLECULE: V-TYPE ATP SYNTHASE ALPHA CHAIN;
16	2lqm-A	5.2	4	60	174	13	MOLECULE: PHO RADA INTEIN;
17	8ubs-A	5.1	3	57	150	9	MOLECULE: NRDJ-1;
18	7qst-A	5.1	3.5	59	361	14	MOLECULE: V-TYPE ATP SYNTHASE SUBUNIT A;
19	7qsu-A	5.1	3.3	60	366	13	MOLECULE: V-ATPASE;
20	5o9j-A	5.1	4.9	65	182	22	MOLECULE: TRANSCRIPTION INITIATION FACTOR IIB,TRANSCRIPTION
**P22 UNK_7**							
No:	Chain	Z	rmsd	lali	nres	%id	PDB Description
1	5h4s-A	6	6.1	60	284	23	MOLECULE: L-RHAMNOSE-BINDING LECTIN;
2	2zx4-B	5.8	2.5	58	195	19	MOLECULE: CSL3;

3	8suf-H	5.7	3.2	60	105	22	MOLECULE: TIR DOMAIN-CONTAINING PROTEIN;
4	2zx2-B	5.7	2.2	55	195	20	MOLECULE: CSL3;
5	2zx3-B	5.7	2.4	56	195	20	MOLECULE: CSL3;
6	2zx1-B	5.7	2.2	55	195	20	MOLECULE: CSL3;
7	2zx0-B	5.7	2.4	56	195	20	MOLECULE: CSL3;
8	8suf-E	5.6	3.6	61	108	21	MOLECULE: TIR DOMAIN-CONTAINING PROTEIN;
9	8suf-F	5.6	3.7	61	108	21	MOLECULE: TIR DOMAIN-CONTAINING PROTEIN;
10	2zx4-A	5.6	2.4	56	195	20	MOLECULE: CSL3;
11	2zx0-A	5.6	2.5	56	195	20	MOLECULE: CSL3;
12	2zx2-A	5.6	2.5	56	195	20	MOLECULE: CSL3;
13	5afb-A	5.6	3	57	347	19	MOLECULE: LATROPHILIN-3;
14	2zx1-A	5.6	2.5	56	195	20	MOLECULE: CSL3;
15	8suf-G	5.5	3.2	60	107	22	MOLECULE: TIR DOMAIN-CONTAINING PROTEIN;
16	6ske-D	5.5	2.6	56	96	23	MOLECULE: TENEURIN-2;
17	6ske-B	5.5	2.6	56	96	23	MOLECULE: TENEURIN-2;
18	5ftt-G	5.4	5.4	63	368	17	MOLECULE: LEUCINE-RICH REPEAT TRANSMEMBRANE PROTEIN FLRT2;
19	2zx3-A	5.4	2.5	56	195	20	MOLECULE: CSL3;
20	8djg-F	5.4	2.9	57	99	19	MOLECULE: SAB HEAVY CHAIN;

P36 UNK_8

No:	Chain	Z	rmsd	lali	nres	%id	PDB Description
1	2w5f-A	7.7	2.6	100	511	9	MOLECULE: ENDO-1,4-BETA-XYLANASE Y;
2	2wze-B	7.7	2.7	102	516	9	MOLECULE: ENDO-1,4-BETA-XYLANASE Y;
3	2w5f-B	7.7	2.7	101	513	9	MOLECULE: ENDO-1,4-BETA-XYLANASE Y;
4	2wys-B	7.6	2.7	101	516	9	MOLECULE: ENDO-1,4-BETA-XYLANASE Y;
5	2wys-A	7.6	2.7	102	513	9	MOLECULE: ENDO-1,4-BETA-XYLANASE Y;
6	1pnf-A	7.6	2.9	95	314	13	MOLECULE: PEPTIDE-N(4)-(N-ACETYL-BETA-D-GLUCOSAMINYL)ASPARA
7	2wze-A	7.5	2.7	102	512	9	MOLECULE: ENDO-1,4-BETA-XYLANASE Y;
8	1uxz-A	7.5	2.8	90	131	12	MOLECULE: CELLULASE B;
9	6za2-A	7.5	3	94	1081	7	MOLECULE: POR SECRETION SYSTEM PROTEIN PORU;
10	1png-A	7.5	2.9	95	310	13	MOLECULE: PEPTIDE-N(4)-(N-ACETYL-BETA-D-GLUCOSAMINYL)

11	1uyx-A	7.4	2.8	89	130	12	MOLECULE: CELLULASE B;
12	1uyy-B	7.4	2.8	90	131	12	MOLECULE: CELLULASE B;
13	1dyo-B	7.4	3	103	156	13	MOLECULE: ENDO-1,4-BETA-XYLANASE Y;
14	4d0q-A	7.4	2.8	100	161	13	MOLECULE: HYALURONATE LYASE;
15	1h6x-A	7.4	2.8	102	159	11	MOLECULE: ENDO-1,4-BETA-XYLANASE Y;
16	6vls-D	7.3	3.1	94	963	12	MOLECULE: MALTOSE/MALTODEXTRIN-BINDING PERIPLASMIC PROTEIN,
17	4xup-D	7.3	2.9	103	321	6	MOLECULE: ENDO-1,4-BETA-XYLANASE C;
18	1uxz-B	7.3	2.8	90	131	12	MOLECULE: CELLULASE B;
19	1uyz-A	7.3	2.8	90	131	12	MOLECULE: CELLULASE B;
20	1uyy-A	7.3	2.8	90	131	12	MOLECULE: CELLULASE B;

Supplementary Table S7. Summary (number, length) of dairy streptococcal phage contigs identified in the assembled phageomes of the whey virome samples. Phageomes were assembled using both Phables and MetAnnotatorX2.

**Virome sample**	**Phables assembly**				**MetAnnotatorX2 assembly**			
	**Strep phage Contigs**	**Average length (bp)**	**Max (bp)**	**Min (bp)**	**Strep phage Contigs**	**Average length (bp)**	**Max (bp)**	**Min (bp)**
**S1**	124	699.8	6966	128	2	7916	8078	7754
**S36**	85	1192	4904	188	5	7826.6	8558	6336
**S86**	130	1049	12709	128	6	9538.8	19990	5033
**S87**	2	2056.5	2107	2006	1	-	13389	-
**S13**	1	-	2438	-	0	-	-	-
**S34**	28	1491	9514	231	1	-	28139	-
**S85**	0	-	-	-	0	-	-	-

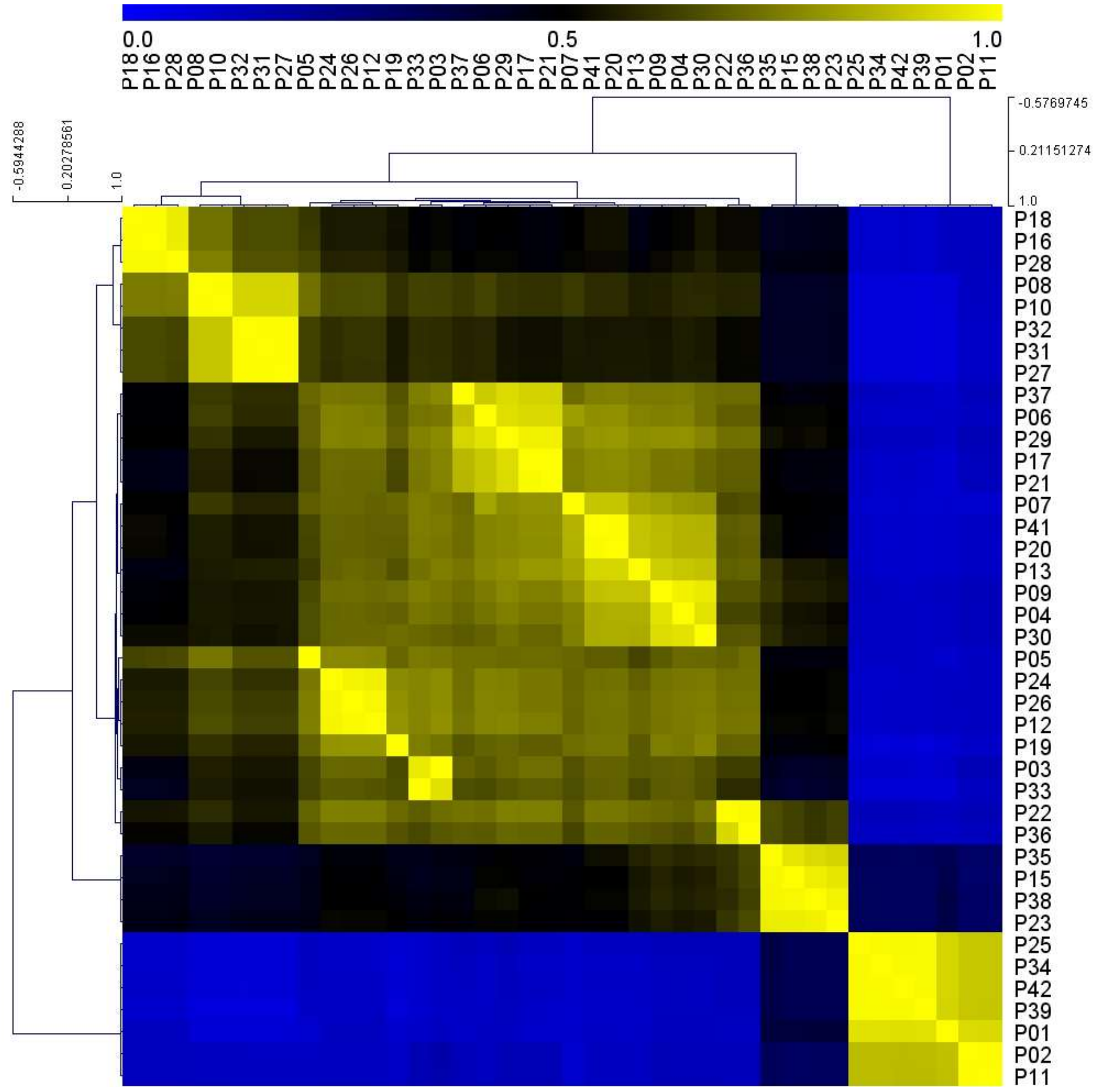

Supplementary Figure S1. Comparative genomic analysis of the sequenced streptococcal phages. Combined heat map indicating the Hadamard score (product of average nucleotide identity and coverage) of each of the 41 streptococcal phages isolated in this study against each other with hierarchical clustering.

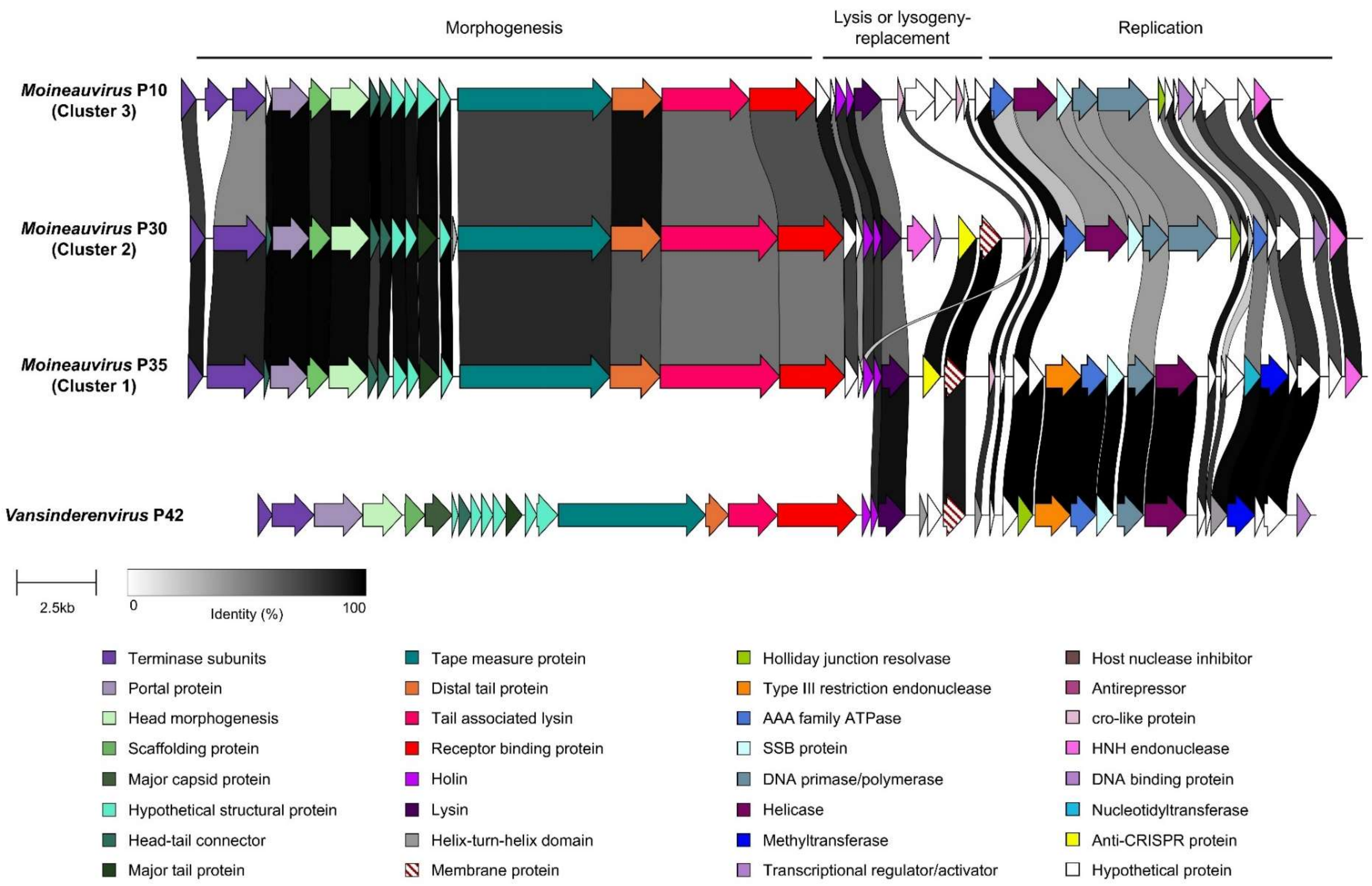

Supplementary Figure S2. Schematic representation of the genetic architecture of a representative of the Vansinderenviruses and each *Moineauvirus* Cluster.

Genome alignments were generated using Clinker (https://cagecat.bioinformatics.nl/tools/clinker).

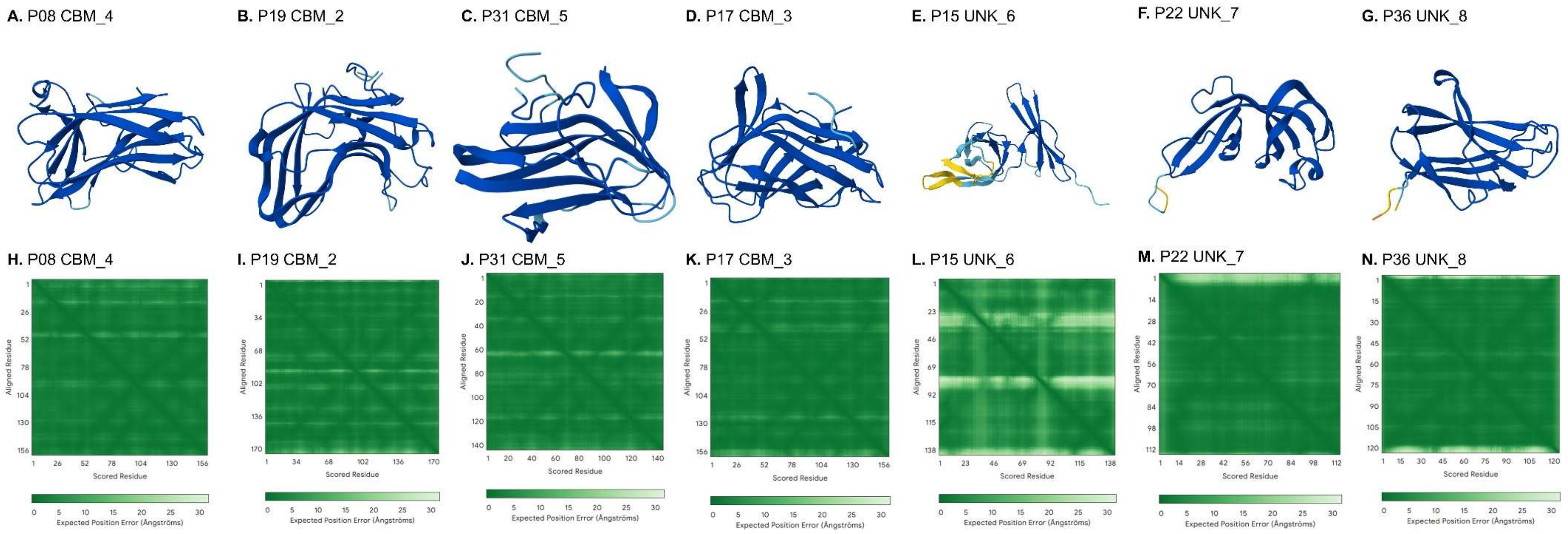

Supplementary Figure S3. Structural predictions of the CBMs and unknown domains (UNK) identified within the Tals of the sequenced Moineauviruses alongside corresponding predicted aligned errors (PAE) plots. A structural prediction of a representative of each CBM and UNK (A-G) is depicted above their associated PAE plots (H-N).

References

1. **Goulet A, Joos R, Lavelle K, Van Sinderen D, Mahony J, *et al.*** A structural discovery journey of streptococcal phages adhesion devices by AlphaFold2. *Front Mol Biosci* 2022;9:960325.
